# Supplementary material for: Integrative proteomics in prostate cancer uncovers robustness against genomic and transcriptomic aberrations during disease progression
Source: Nat Commun. 2018 Mar 21;9:1176. doi: 10.1038/s41467-018-03573-6 (PMC5862881; doi:10.1038/s41467-018-03573-6)
Supplement: Supplementary file 1 — Supplementary Information(PDF 2259 kb) [file 41467_2018_3573_MOESM1_ESM.pdf]

Supplementary Information for:

Integrative proteomics in prostate cancer uncovers robustness against genomic and transcriptomic aberrations during disease progression

Latonen et al.

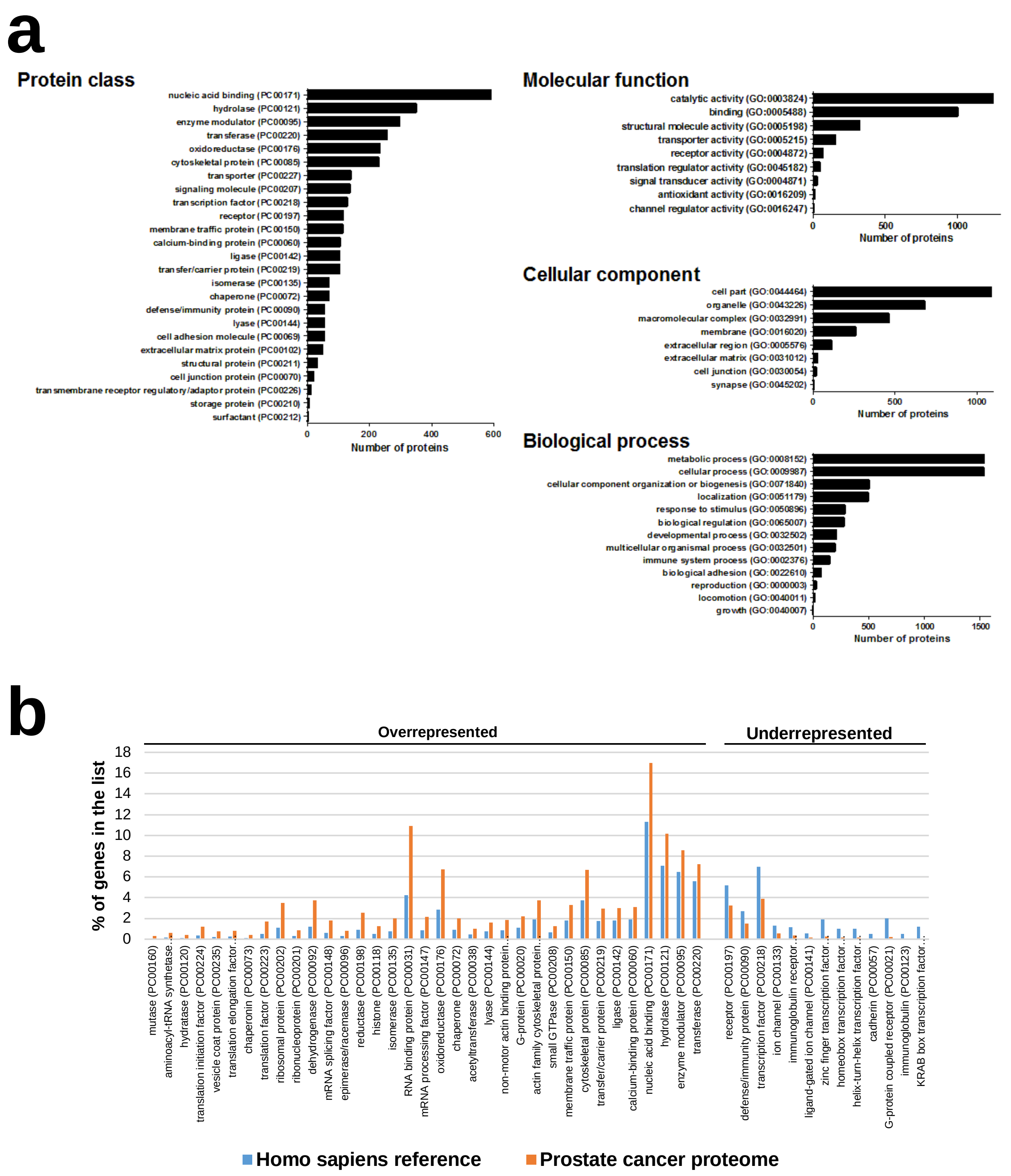

Supplementary Figure 1. Classification of proteins identified in the mass spectrometric analysis. **a**) Major protein classes of the proteins identified in the BPH, PC, and CRPC samples by the SWATH-MS analysis. Proteins are grouped based on their major protein class (PANTHER protein class) and gene ontology groups (GO; molecular functions, cellular components, and biological processes). **b)** The over- and underrepresented protein classes and subclasses as compared to *Homo sapiens* reference list according to PANTHER analysis. It must be noted that a single protein can belong to several of the presented groups and subgroups (e.g. proteins in the group of homeobox transcription factors belong also to the groups of helix-turn-helix transcription factors and transcription factors).

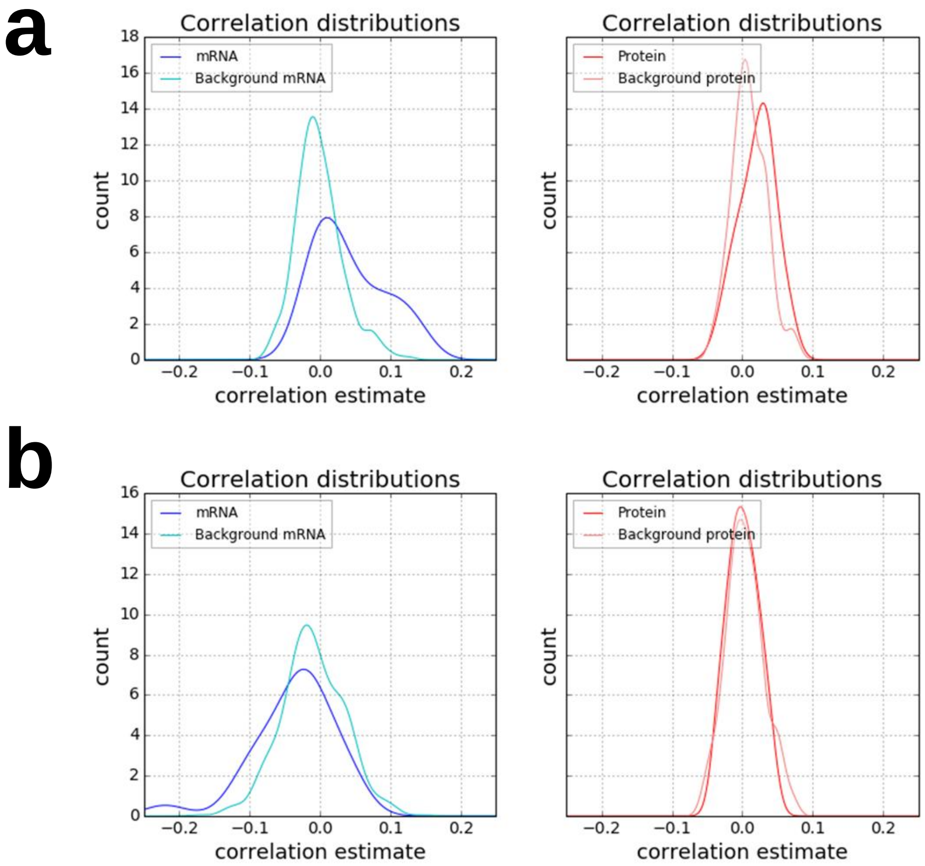

Supplementary Figure 2. Observed global correlation patterns are driven biologically as opposed to mere chance. a) left panel: correlation distribution of mRNA expression and gene copy number and its null distribution; right panel: correlation distribution of protein expression and gene copy number and its null distribution. **b)** left panel: correlation distribution of mRNA and DNA methylation and its null distribution; right panel: correlation distribution of protein expression and DNA methylation and its null distribution.

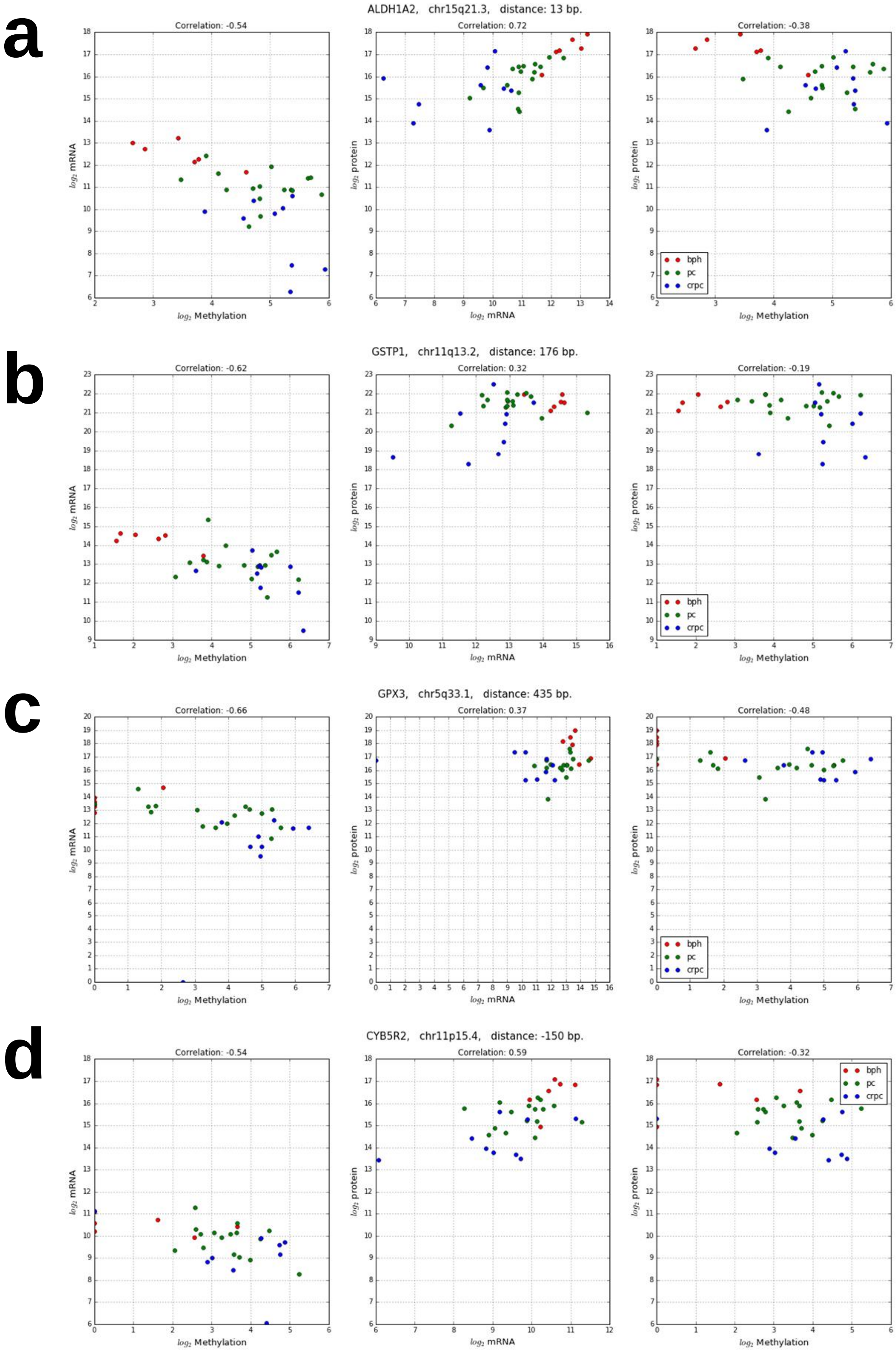

Supplementary Figure 3. Examples of previously known genes whose DNA methylation is increased in prostate cancer, correlating with decreased expression at the RNA and protein levels. Correlation between mRNA expression level and DNA methylation (left panels), protein expression and mRNA expression levels (middle panels) and protein expression and DNA methylation levels (right panels) of **a)** ALDH1A2, **b)** GSTP1, **c)** GPX3, and **d)** CYB5R2. Chromosome locations of the genes, and the DMR distance from TSS are shown. Correlation values are shown above each graph.

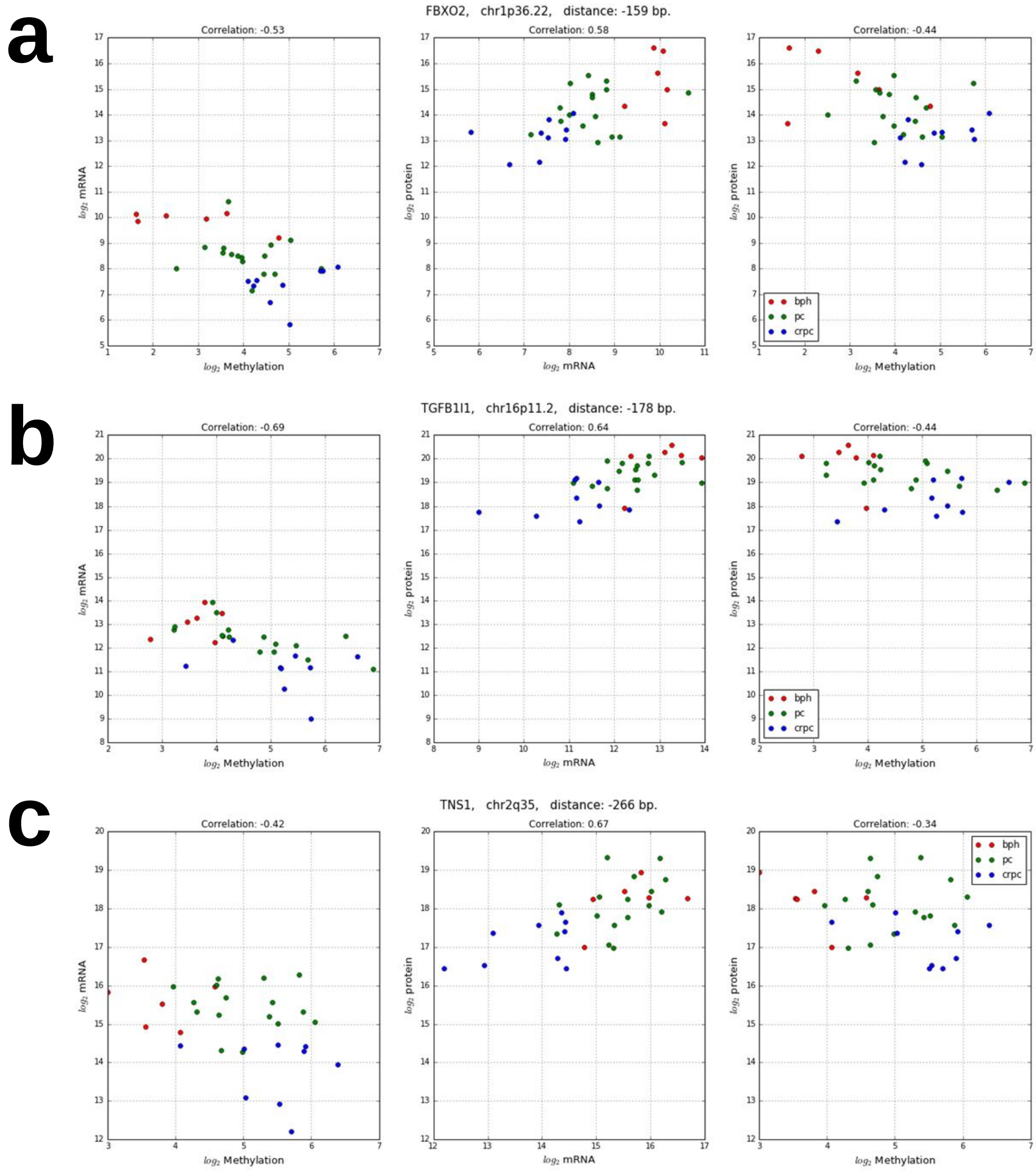

Supplementary Figure 4. Examples of genes whose promoter DNA methylation is increased in prostate cancer, correlating with decreased expression at the RNA and protein levels. Correlation between mRNA expression level and DNA methylation (left panels), protein expression and mRNA expression levels (middle panels) and protein expression and DNA methylation levels (right panels) of **a)** FBXO2, **b)** TGFB1I1, and **c)** TNS1. Chromosome locations of the genes, and the DMR distance from TSS are shown. Correlation values are shown above each graph.

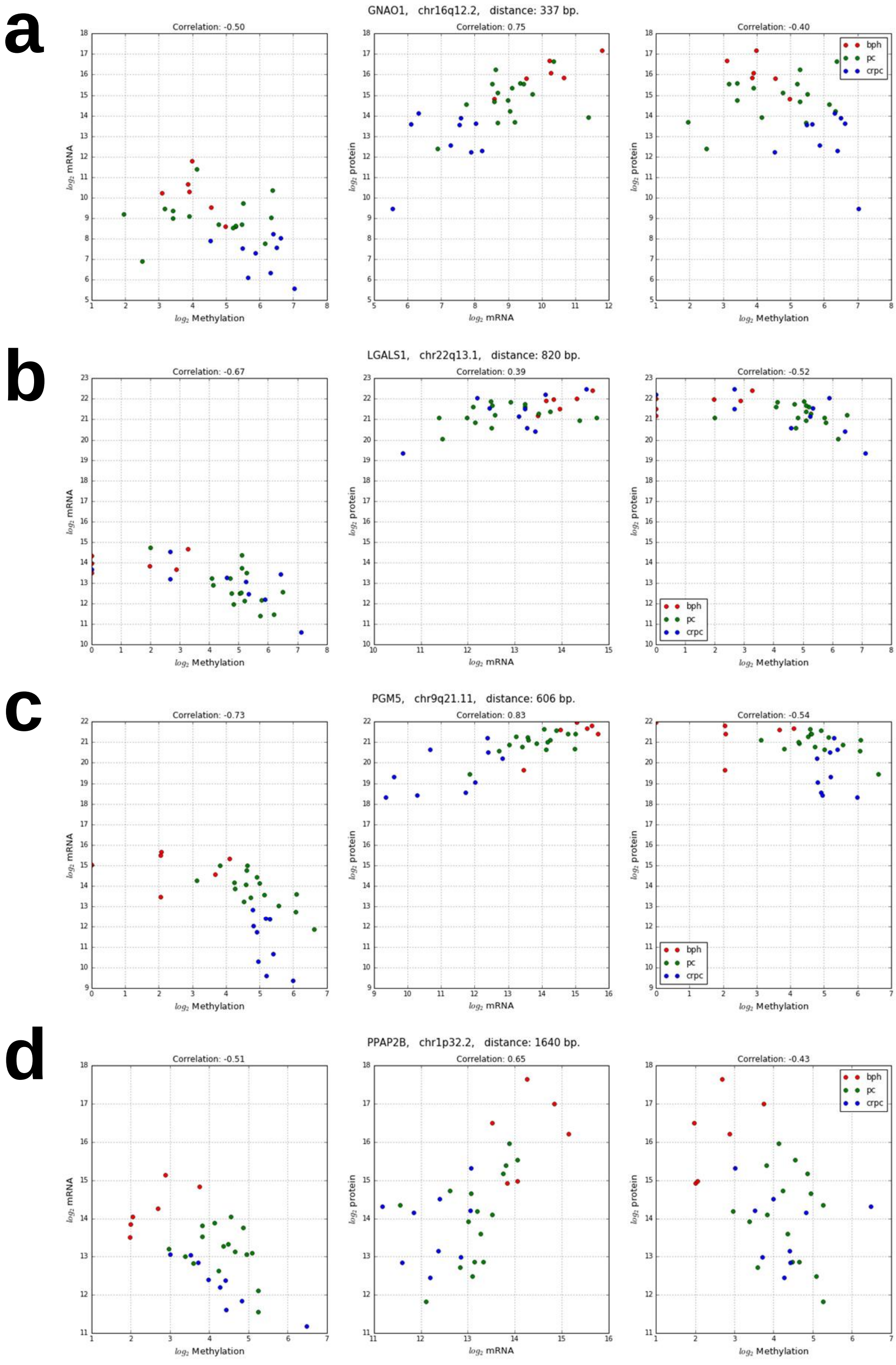

Supplementary Figure 5. Examples of genes whose gene body DNA methylation is increased in prostate cancer, correlating with decreased expression at the RNA and protein levels. Correlation between mRNA expression level and DNA methylation (left panels), protein expression and mRNA expression levels (middle panels) and protein expression and DNA methylation levels (right panels) of **a)** GNAO1, **b)** LGALS1, **c)** TNS1, and **d)** PPAP2B. Chromosome locations of the genes, and the DMR distance from TSS are shown. Correlation values are shown above each graph.

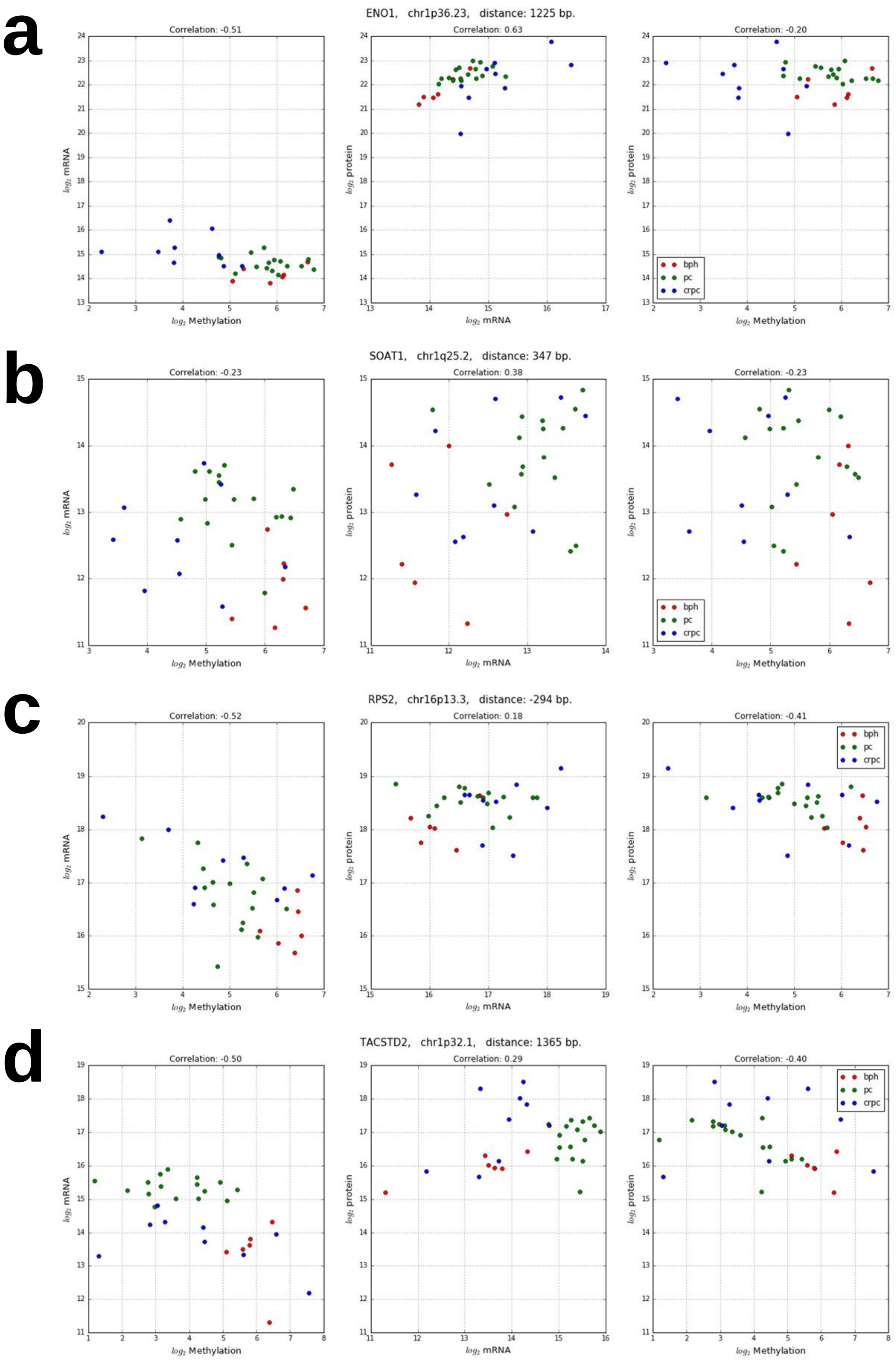

Supplementary Figure 6. Examples of genes whose DNA methylation is decreased in prostate cancer, correlating with increased expression at the RNA and protein levels. Correlation between mRNA expression level and DNA methylation (left panels), protein expression and mRNA expression levels (middle panels) and protein expression and DNA methylation levels (right panels) of **a)** ENO1, **b)** SOAT1, **c)** RPS2, and **d)** TACSTD2. Chromosome locations of the genes, and the DMR distance from TSS are shown. Correlation values are shown above each graph.

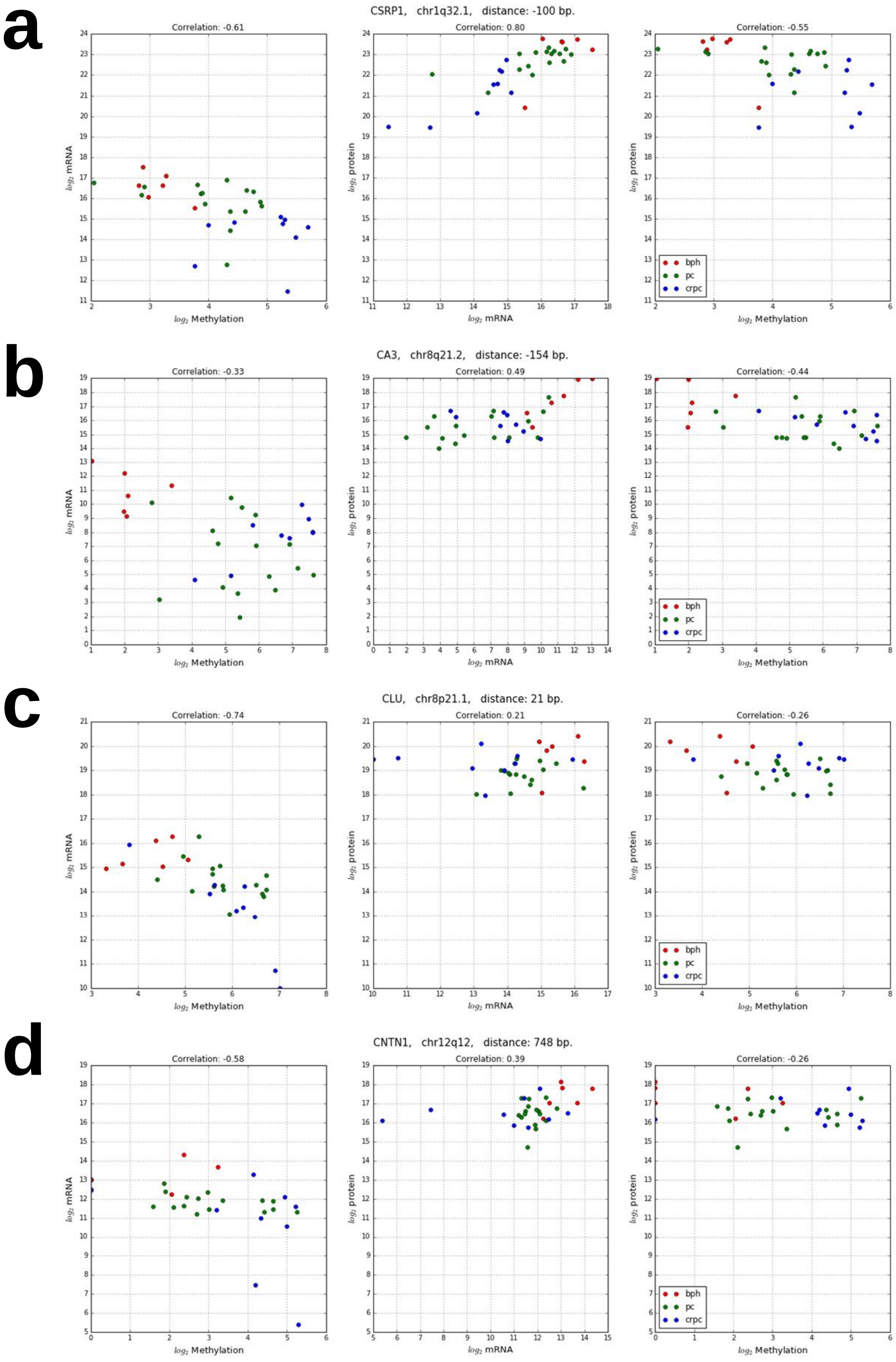

Supplementary Figure 7. Examples of genes whose DNA methylation is increased in prostate cancer samples without an effect to protein levels in cancer cells. Correlation between mRNA expression level and DNA methylation (left panels), protein expression and mRNA expression levels (middle panels) and protein expression and DNA methylation levels (right panels) are shown for **a)** CSRP1, and **b)** CA3 expressed in stromal cells, as well as **c)** CLU, and **d)** CNTN1, protein expression of which does not correlate with increased DMR methylation or decreased RNA methylation. Chromosome locations of the genes, and the DMR distance from TSS are shown. Correlation values are shown above each graph.

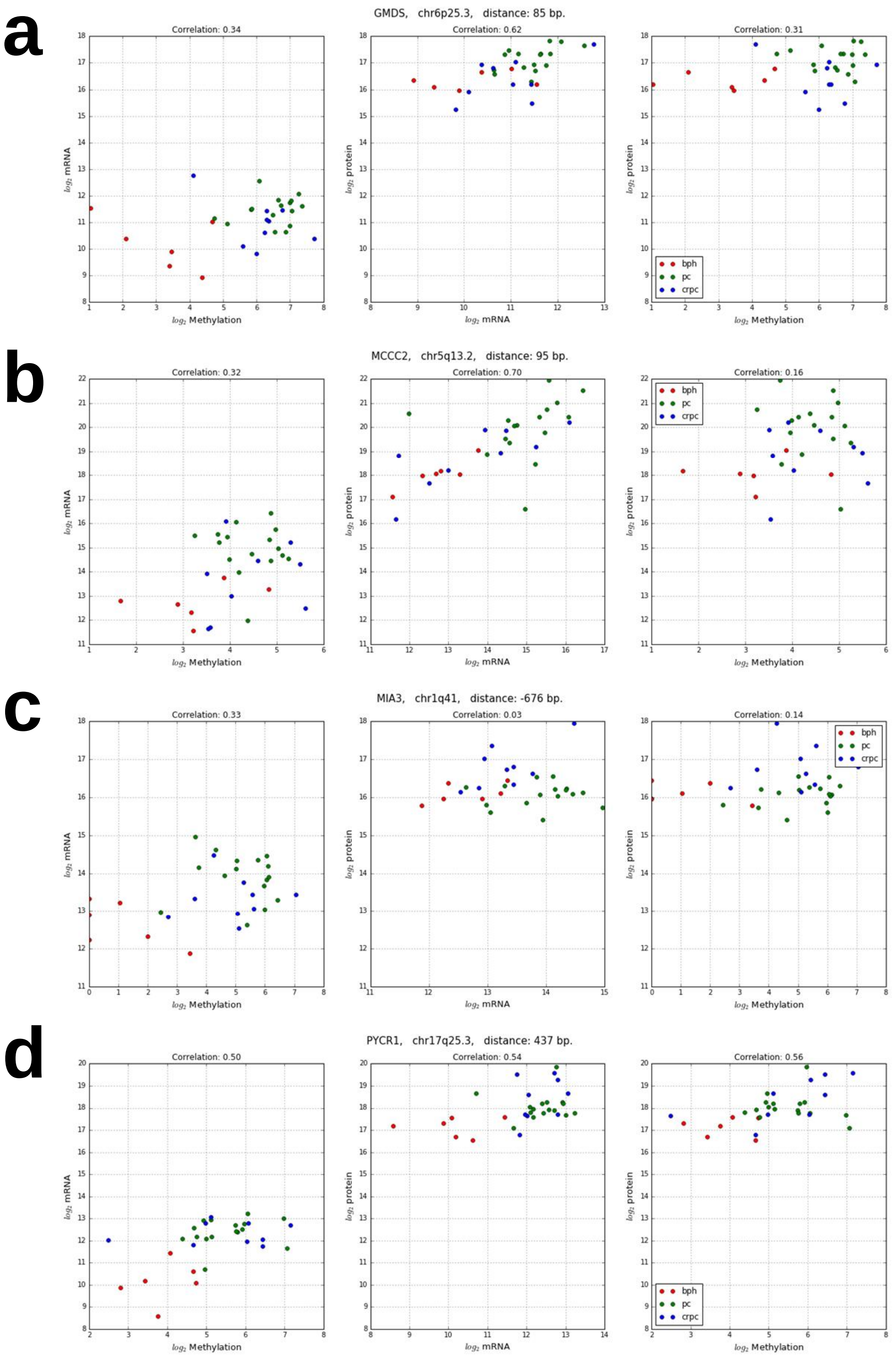

Supplementary Figure 8. Examples of genes whose DNA methylation is increased in prostate cancer, correlating with increased expression at the RNA and protein levels. Correlation between mRNA expression level and DNA methylation (left panels), protein expression and mRNA expression levels (middle panels) and protein expression and DNA methylation levels (right panels) of **a)** GMDS, **b)** MCCC2, **c)** MIA3, and **d)** PYCR1. Chromosome locations of the genes, and the DMR distance from TSS are shown. Correlation values are shown above each graph.

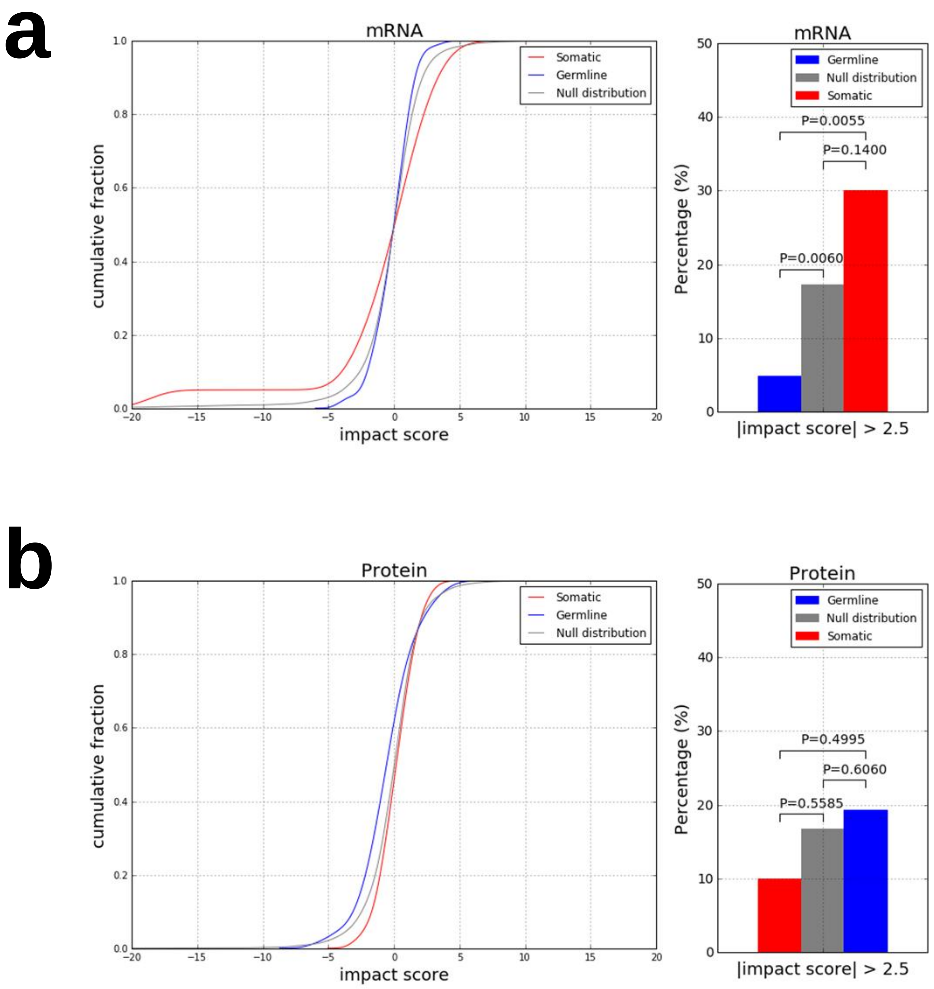

Supplementary Figure 9. Impact of somatic and germline variants on mRNA and protein expression in prostate cancer. a) Impact of mutations to mRNA expression levels and **b)** protein expression levels. Impact scores of somatic and germline variants compared to null distributions are shown in the panels on the left. Percentages of variants with absolute impact score above 2.5 and significances between mutation groups assessed by Fisher's exact test are shown in the panels on the right.

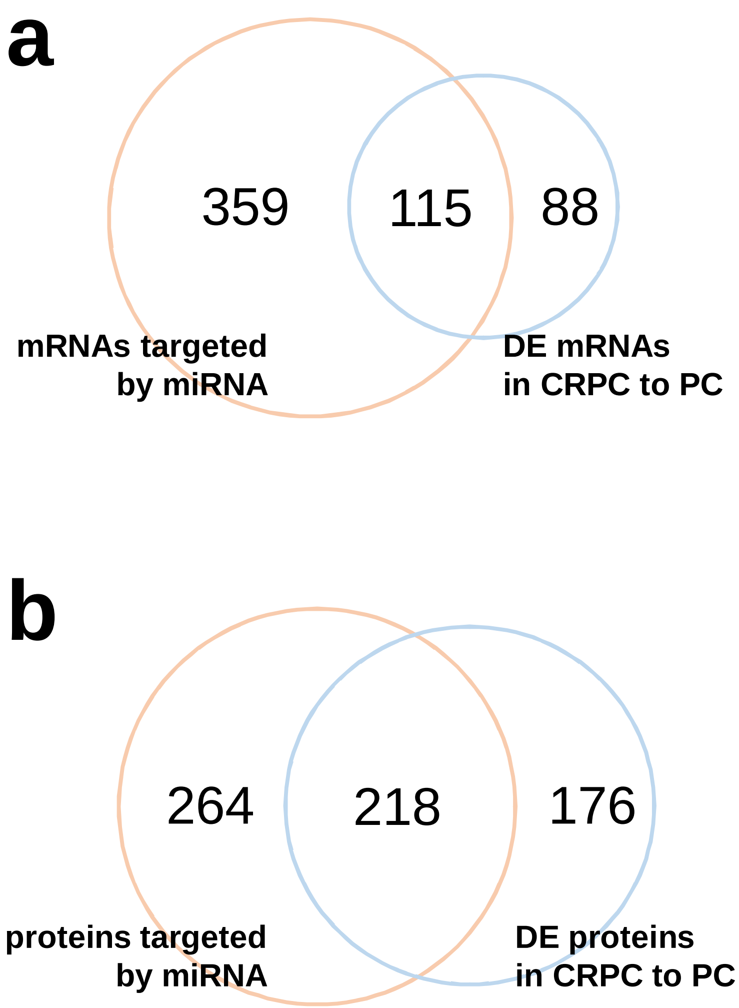

Supplementary Figure 10. Analysis of miRNA targets from differentially expressed pools of mRNA and protein during prostate cancer progression. a) Overlap between mRNAs that are potential targets of an negatively correlating miRNA, and the pool of differentially expressed mRNAs in CRPC to PC comparison. The overlapping 115 genes represent the pool of genes that may be regulated by the targeting miRNAs via mRNA degradation during prostate cancer progression. **b)** Overlap between proteins that are potential targets of an negatively correlating miRNA, and the pool of differentially expressed proteins in CRPC to PC comparison. The overlapping 218 genes represent the pool of genes that may be regulated by the targeting miRNAs via inhibition of translation (i.e. w/o mRNA degradation) during prostate cancer progression.

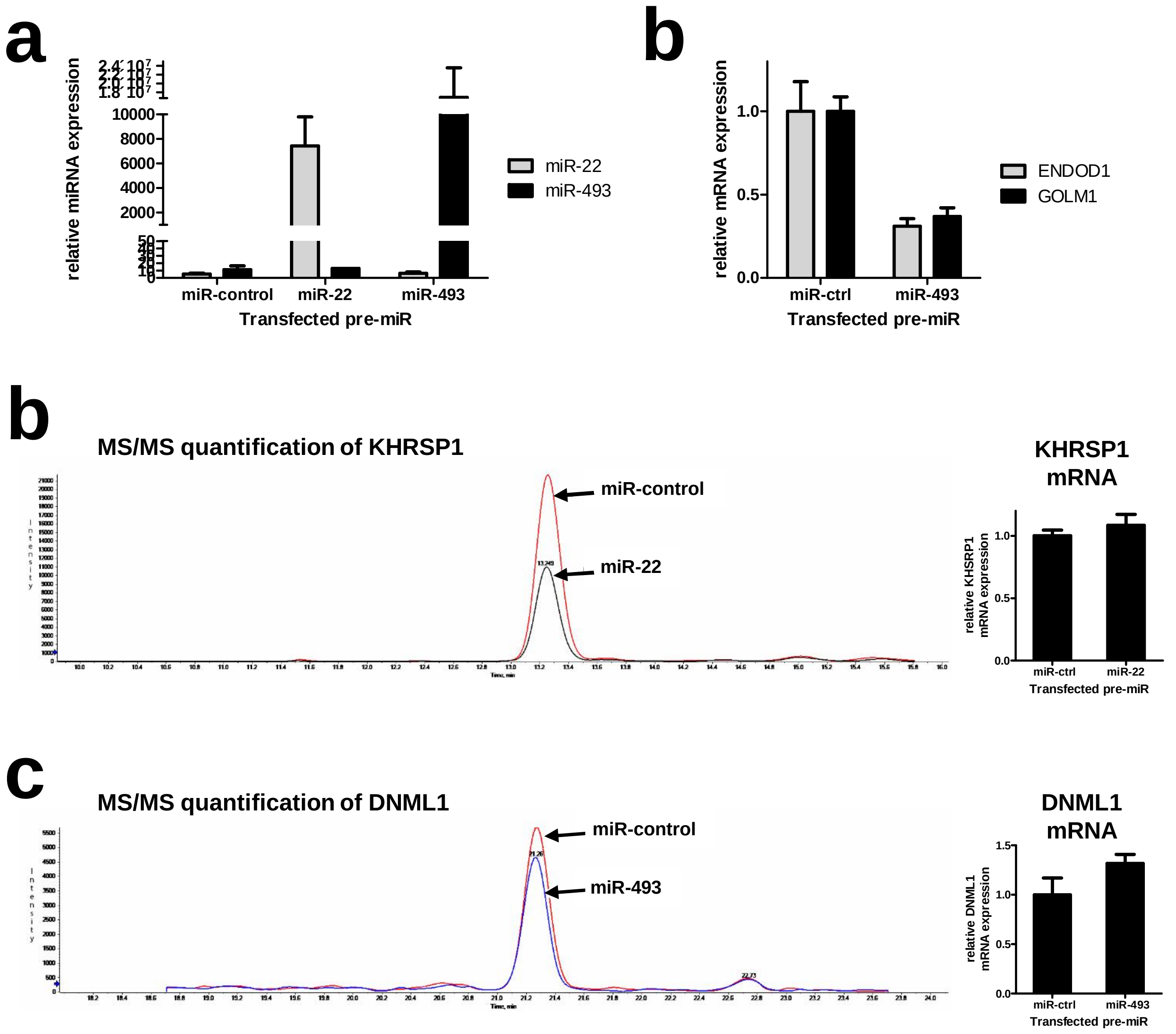

Supplementary Figure 11. Verification of predicted miRNA-target pairs found significantly regulated in prostate cancer. Examples of predicted miRNA-target pairs having negative correlation based on either transcriptomic or proteomic analyses were verified by assessing mRNA and protein levels of the targets in PC-3 cells transfected with pre-miRNA constructs. **a)** Successful transfections and expressions of the transfected miRNAs were verified by TaqMan microRNA RT-qPCR assays. **b)** Expression of two predicted miR-493 targets, ENDOD1 and GOLM1, which showed negative correlations with miR-493 expression intranscriptome-based analysis, were found to be significantly decreased at the mRNA level by increased expression of miR-493 as assessed by RT-qPCR analysis. **c)** Expression of a predicted miR-22 target, KHSRP1, which showed inverse correlation with miR-22 expression in proteomic but not in transcriptomic analysis, was verified to be significantly decreased at the protein level by MicroLC-MSTRAP (MS/MS quantification; left panel) but not at the mRNA level by RT-qPCR analysis (right panel). **d)** Expression of a predicted miR-493 target, DNML1, which showed negative correlation with miR-493 expression in proteomic but not in transcriptomic analysis, was verified to be significantly decreased at the protein level by MicroLC-MSTRAP (MS/MS quantification; left panel) but not at the mRNA level by RT-qPCR analysis (right panel). Error bars in all panels respresent S.D. between three technical replicates.

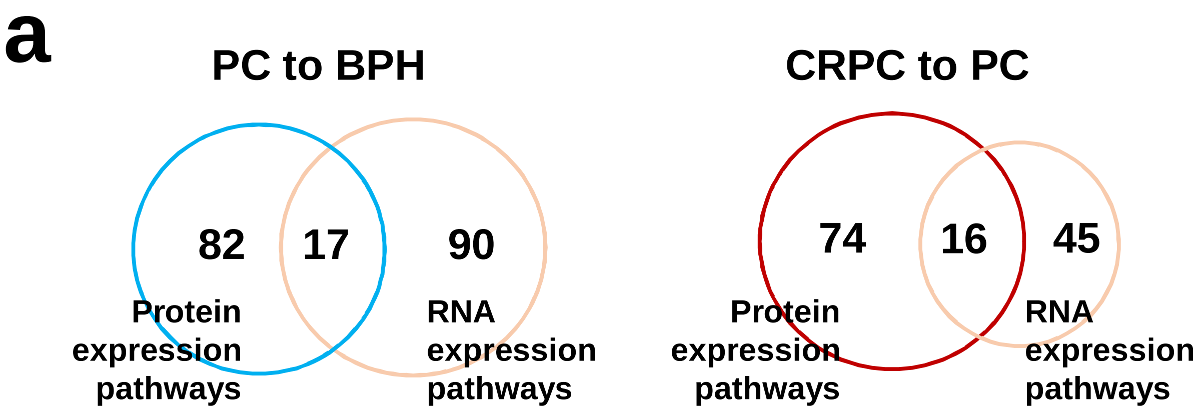

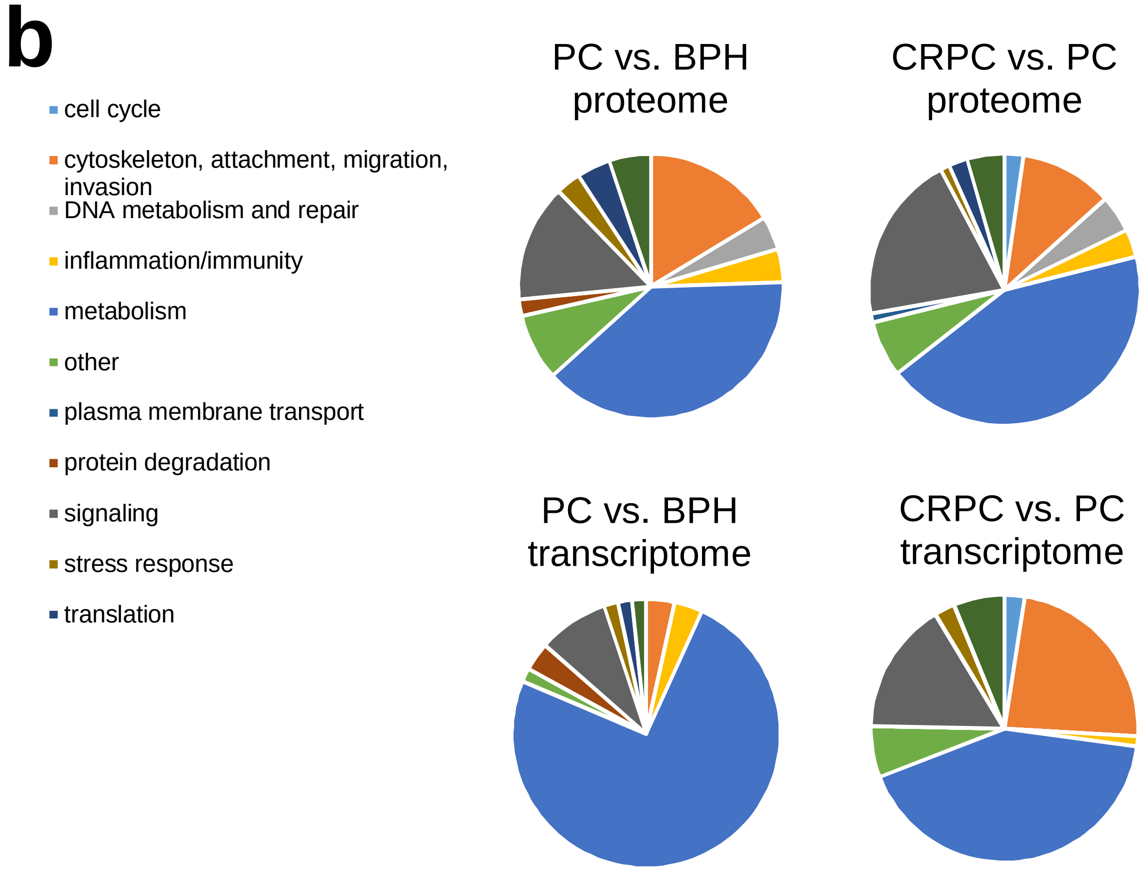

Supplementary Figure 12. Comparison of pathways identified as differentially regulated in prostate cancer based on mRNA expression according to RNA sequencing and protein expression according to mass spectrometric analyses. A) Pie charts of groups of pathways identified as differentially regulated by proteomics and RNA sequencing in PC to BPH and CRPC to PC comparisons. **B)** Overlap between pathways identified as differentially regulated in proteomic and RNA sequencing data in PC to BPH and CRPC to PC comparisons.

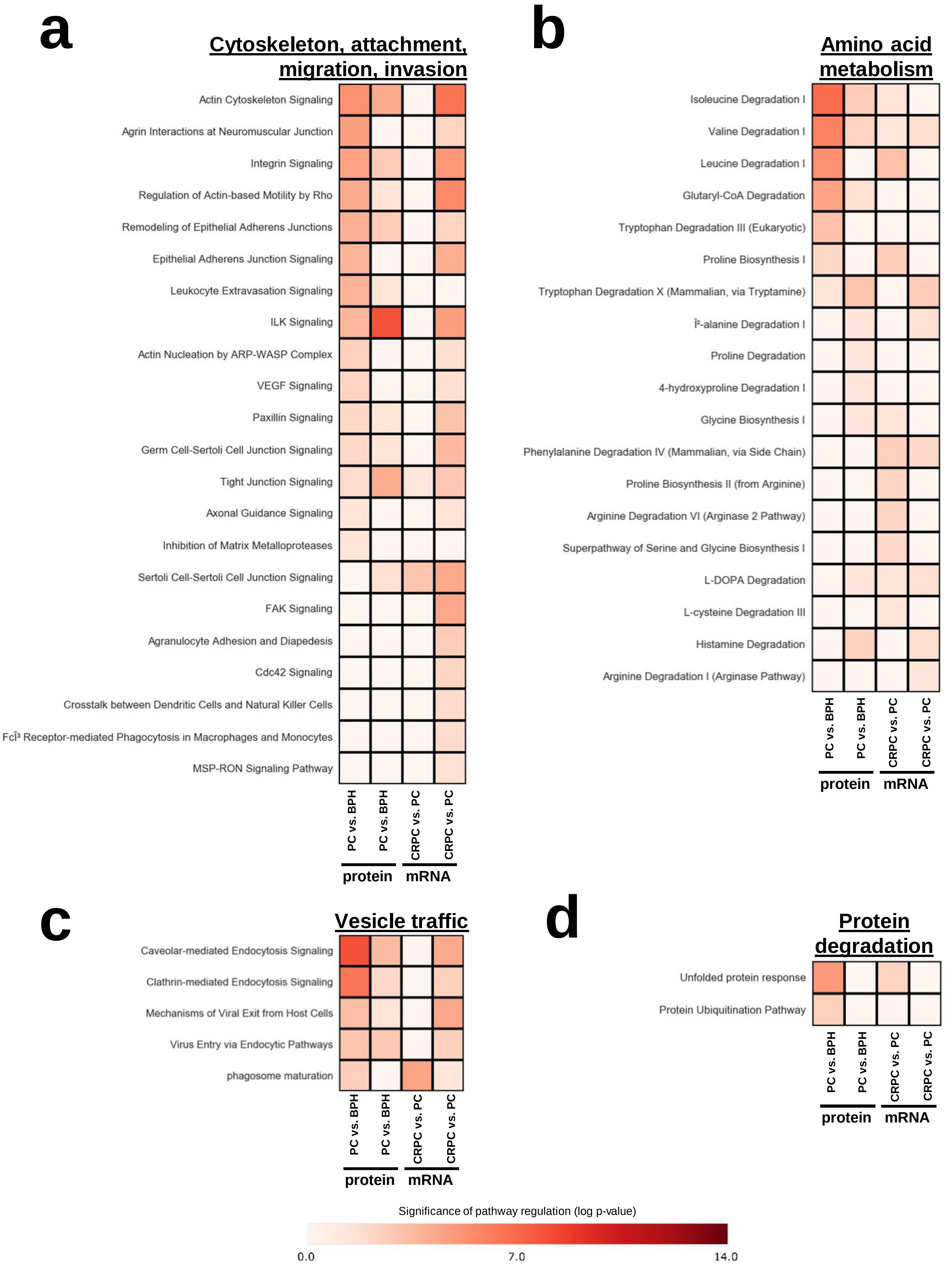

Supplementary Figure 13. Examples of differentially expressed pathways in prostate cancer. Pathways identified as differentially regulated based on mRNA expression (m) according to RNA sequencing and protein expression (p) according to mass spectrometric analyses in PC vs. BPH and CRPC vs. PC comparisons. **A)** Pathways related to cytoskeleton, attachment, migration and invasion. **B)** Amino acid metabolism pathways. **C)** Vesicle traffic-related pathways. **D)** Protein degradation-related pathways. The color key below applies to all panels.

TCA proteins considered altered in IPA pathway analysis

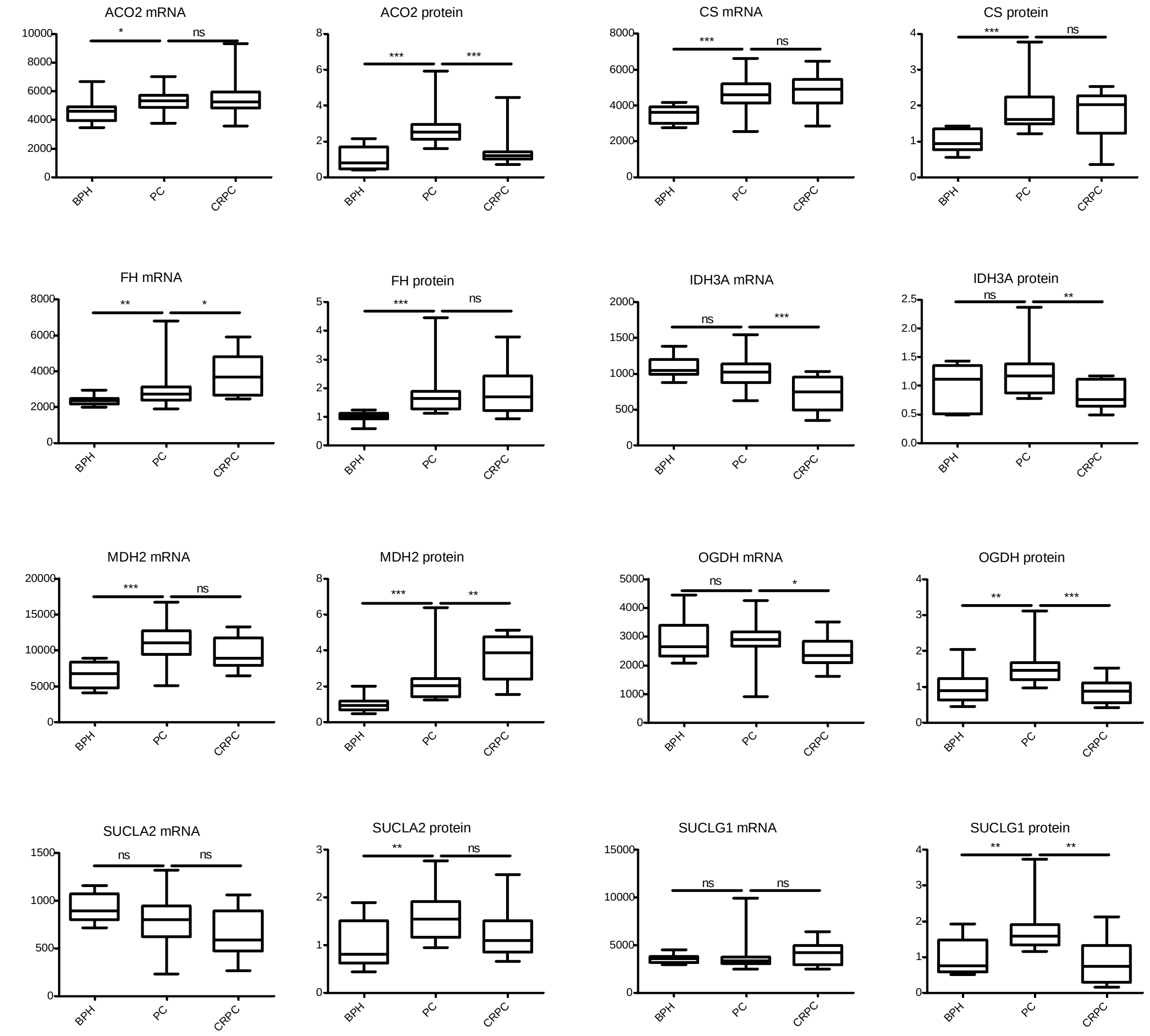

TCA proteins considered not altered in IPA pathway analysis

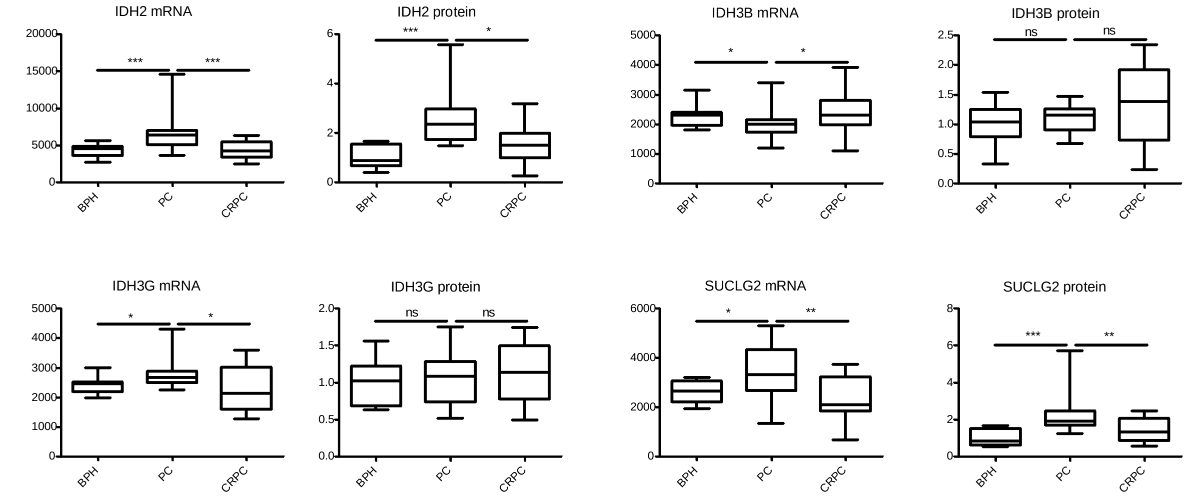

Supplementary Figure 14. Expression of TCA cycle genes at mRNA and protein levels. mRNA (RNAseq; left panels) and protein (mass spectrometry; right panels) levels in BPH, PC and CRPC samples. Boxplots show interquartiles with mean values, whiskers represent minimum and maximum values. Significances between groups are shown (*p<0.05, **p<0.01, ***p<0.001, ns=not significant; Mann-Whitney test).

Protein: ACO2
Peptide: SQFTITPGSEQIR

BPH_718
Intensity: 500

BPH_719
Intensity: 800

PC_470
Intensity: 9 000

PC_4786
Intensity: 10 000

CRPC_530
Intensity: 3700

CRPC_679
Intensity: 800

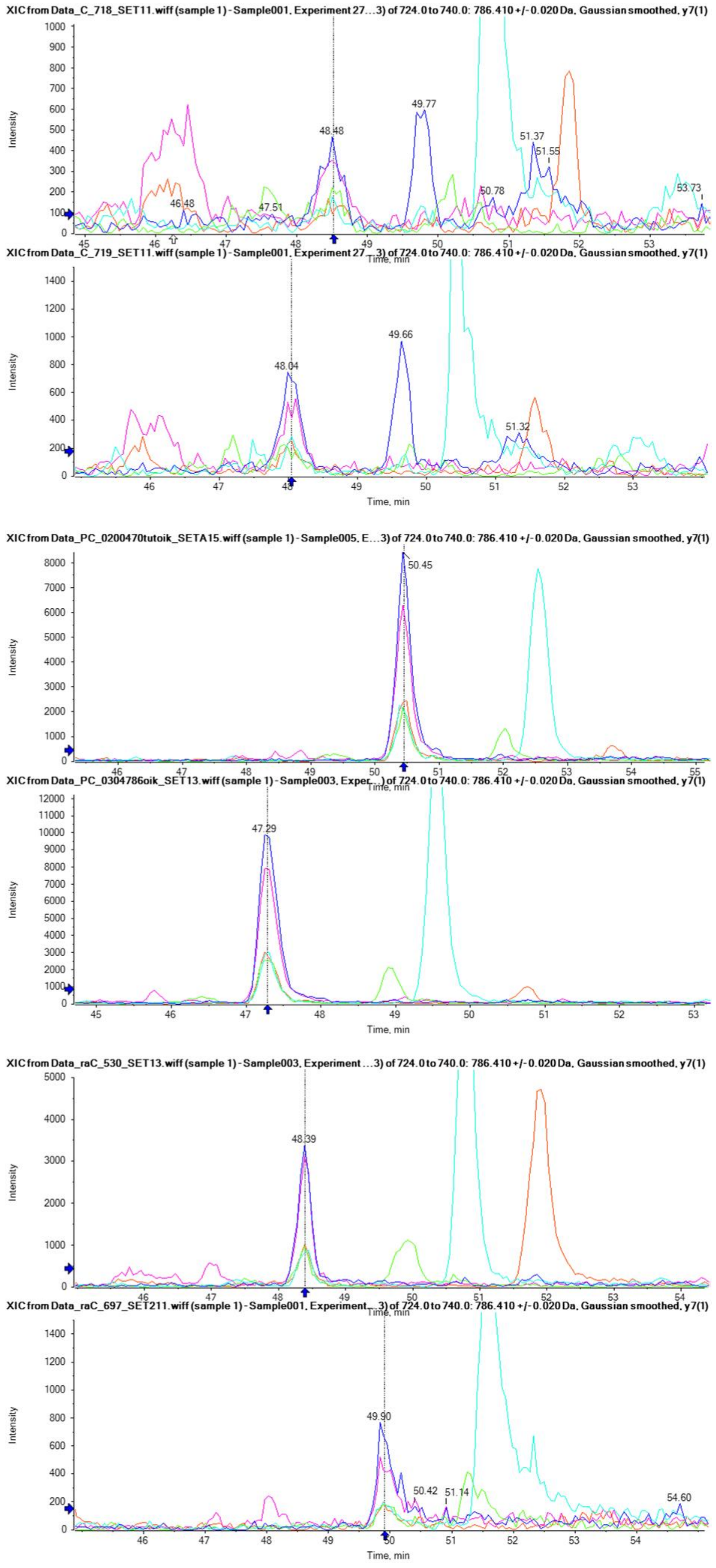

Supplementary Figure 15. Mass spectrometry quantification of ACO2 protein in prostate cancer. Total ion chromatograms for ACO2 peptide SQFTITPGSEQIR for selected samples used also in discovery MS and Western analyses. The fragment intensities are shown. It must be noted that the graphs are on different scales.

Protein: MDH2
Peptide: EGVVEC[CAM]SFVK

BPH_718
Intensity: 1 800

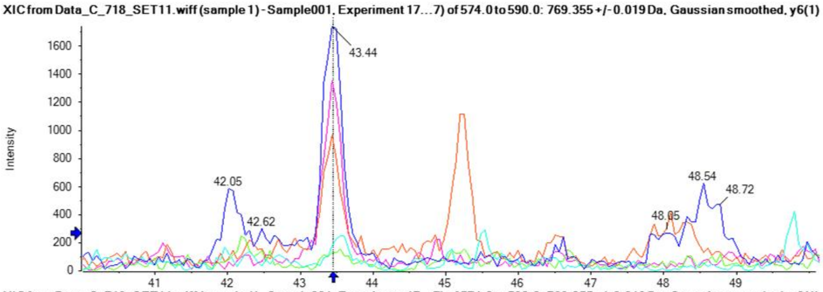

BPH_719
Intensity: 3 700

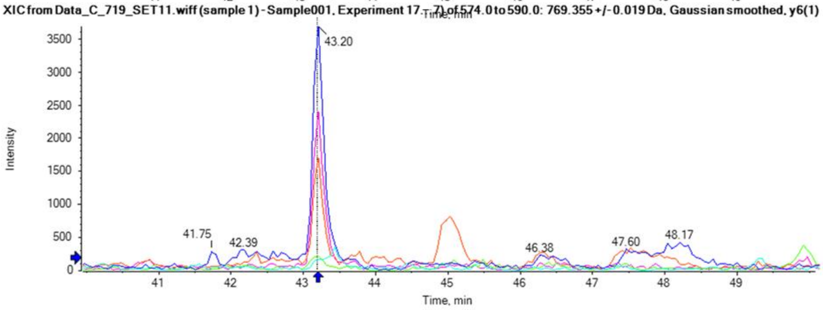

PC_470
Intensity: 22 000

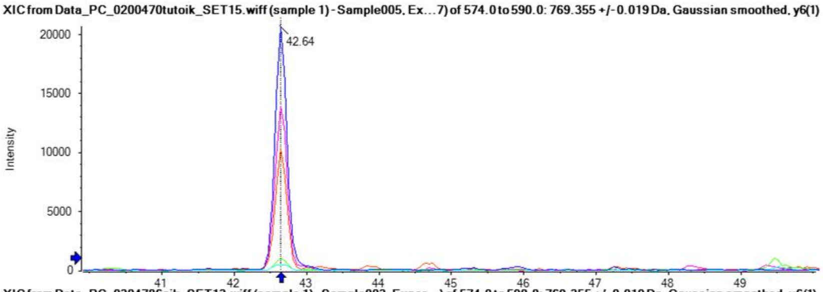

PC_4786
Intensity: 24 500

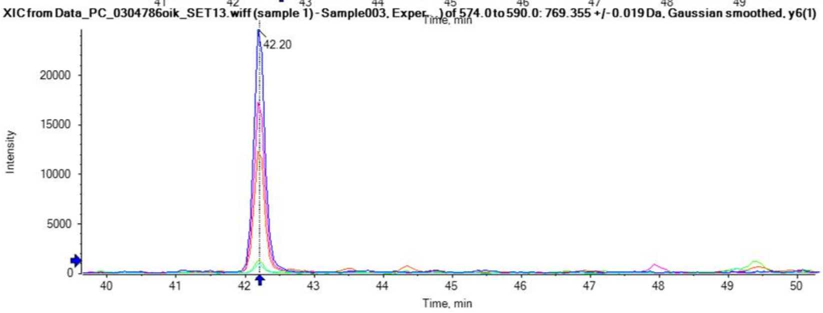

CRPC_530
Intensity: 28 000

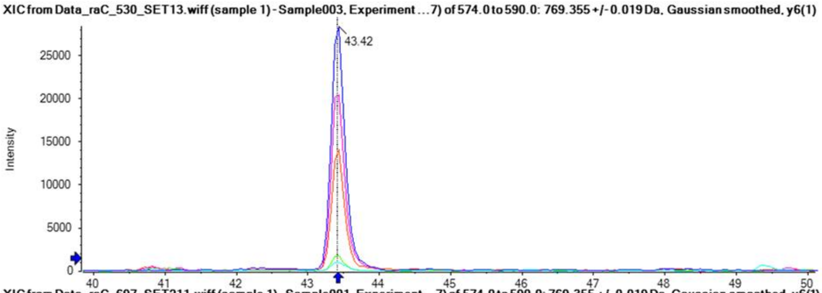

CRPC_697
Intensity: 27 500

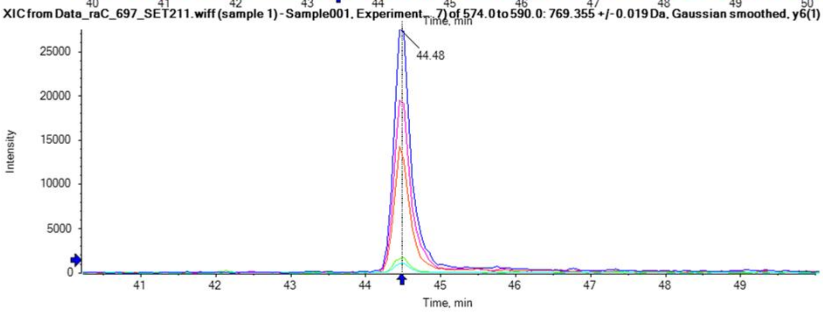

Supplementary Figure 16. Mass spectrometry quantification of MDH2 protein in prostate cancer. Total ion chromatograms for MDH2 peptide EGVVEC[CAM]SFVKfor selected samples used also in discovery MS and Western analyses. The fragment intensities are shown. It must be noted that the graphs are on different scales.

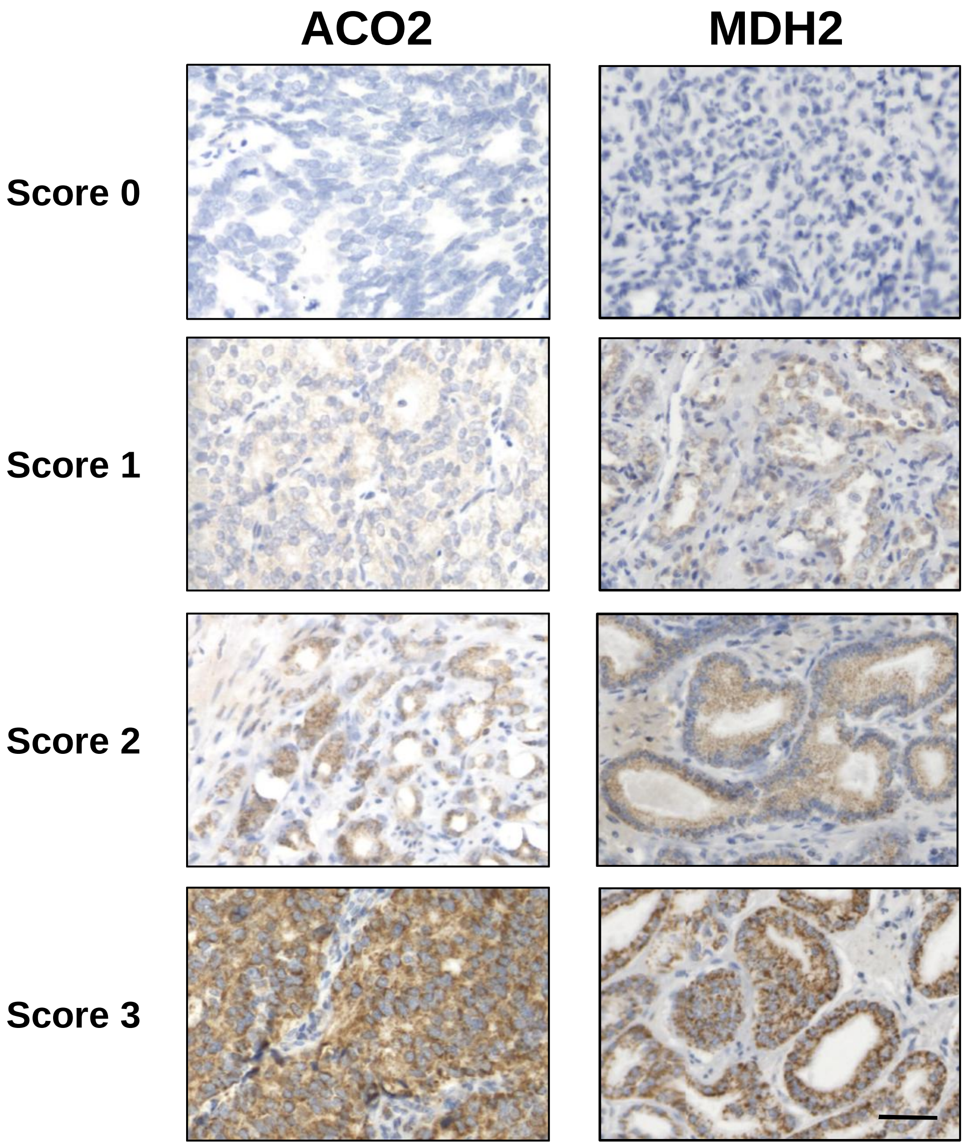

Supplementary Figure 17. Staining intensity scores in immunohistochemical analysis of ACO2 and MDH2. Examples of staining intensities scored as 0,1,2, and 3 are shown for ACO2 (left panel) and MDH2 (right panel). Scale bar 50 ㎛.

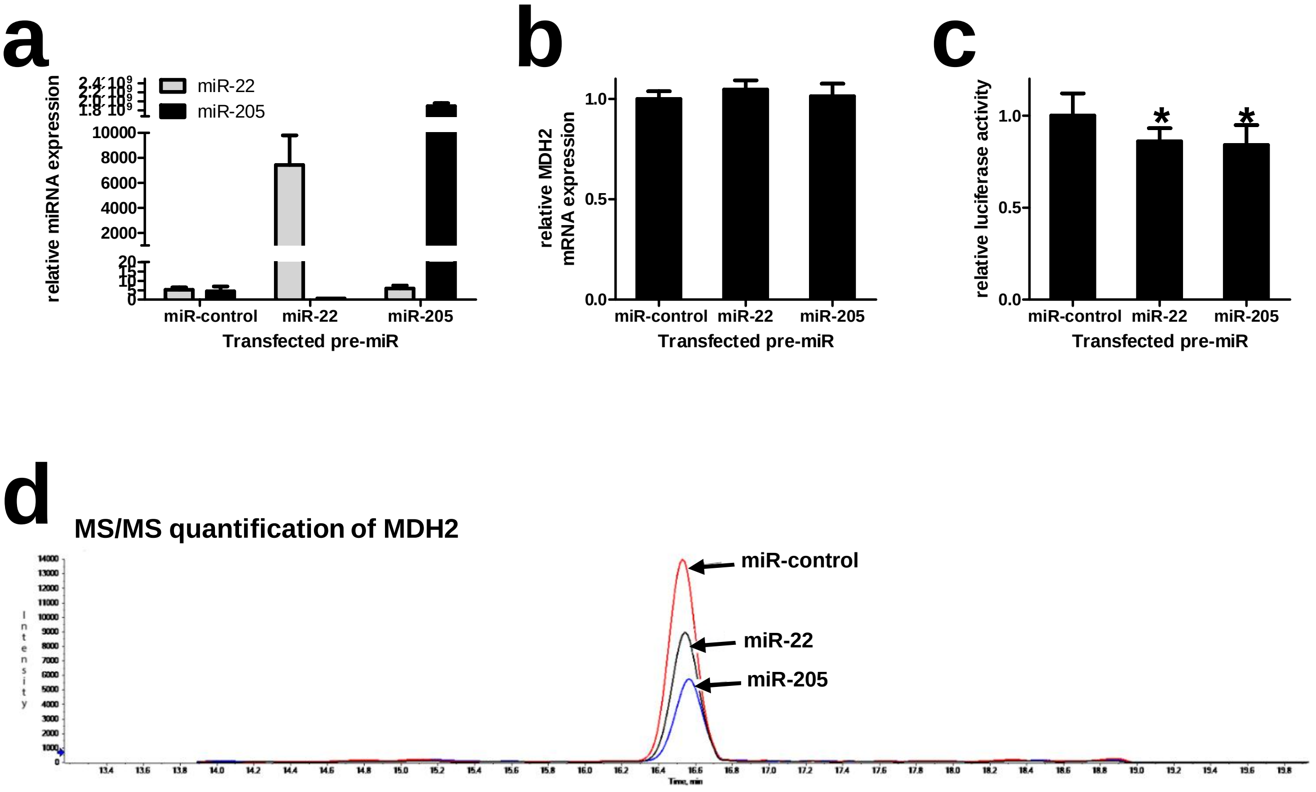

Supplementary Figure 18. MDH2 is targeted by differentially expressed miRNAs in prostate cancer. miR-22 and miR-205, predicted to target MDH2, have decreased expression in CRPC, and negative correlation with MDH2 based on proteomic but not transcriptomic analyses. The ability of these miRNAs to target MDH2 was verified by assessing mRNA and protein levels of MDH2 in PC-3 cells transfected with pre-miRNA constructs. **a)** Successful transfections and expressions of the transfected miRNAs were verified by TaqMan microRNA RT-qPCR assays. Error bars represent S.D. between three technical replicates. **b)** Expression of MDH2 mRNA levels remain unaltered by increased expression of miR-22 and miR-205 as assessed by RT-qPCR analysis. Error bars represent S.D. between three technical replicates. **c)** Increased expression of miR-22 and miR-205 decrease MDH2-3'UTR luciferase reporter activity in pre-miR transfected cells in a statistically significant (*$p<0.05$; unpaired t-test compared to miR-control) manner. Error bars represent S.E.M. between four technical replicates. **d)** Expression of MDH2 was verified to be significantly decreased by increased expression of miR-22 and miR-205 at the protein level as assessed with MicroLC-MSTRAP MS/MS quantification.

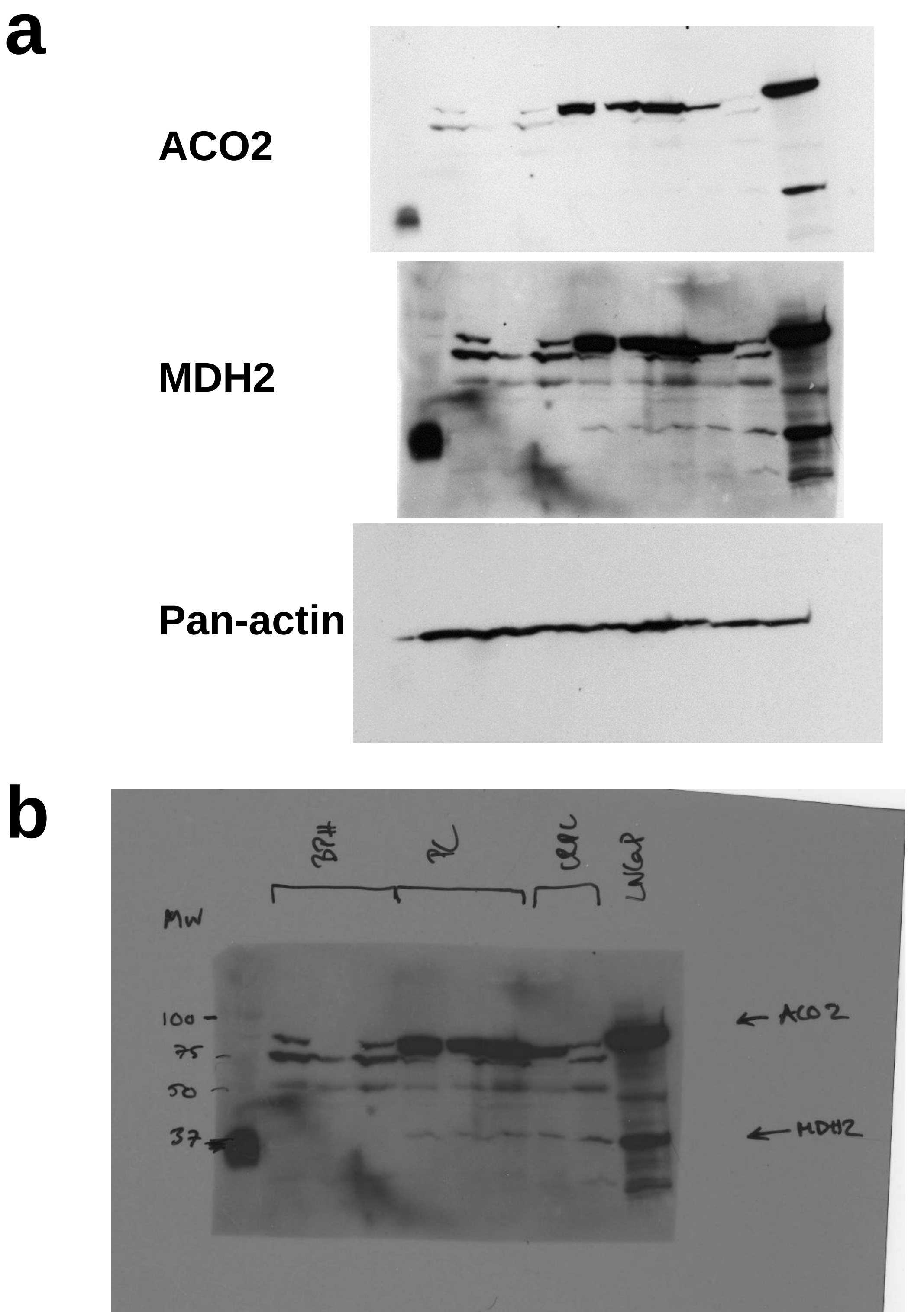

Supplementary Figure 19. Original scans and molecular weight markers for western blots shown in Figure 5. a) Original scans for western blots shown in Figure 5C. **b)** A scan of ACO2 and MDH2 western blot showing molecular weight markers of 37-100kDa. The sizes of the proteins are approximately 85kDa and 35.5kDa for ACO2 and MDH2, respectively. MDH2 is known to run slightly above the expected size.

Supplementary Table 1. Description of primary PC samples used in the study.

Sample ID	Age at diagnosis	PSA at diagnosis	T class	N class	M class	Gleason score	Progression	Progression PSA
PC_5934	67,4	9,6	T1c	NX	M0	6	No	
PC_8131	61.8	6.7	T1c	NX	MX	5	No	
PC_12517	65,5	5,9	T1c	NX	MX	7	No	
PC_15420	60,3	12,6	T1c	NX	M0	7	Yes	0,8
PC_15760	64,7	14	T2	NX	M0	7	Yes	0,8
PC_18307	47,4	5,1	T2	NX	MX	7	No	
PC_9324	67,3	10,3	T2	NX	M0	6	No	
PC_14670	71,8	7,5	T2	NX	MX	8	Yes	2,1
PC_17447	65,8	13,2	T2	NX	M0	9	Yes	1,7
PC_470	70,9	17,6	T2a	NX	M0	8	Yes	
PC_10286	67,8	5,4	T1c	NX	M0	6	No	
PC_15194	62,7	9,5	T2	NX	MX	7	Yes	1
PC_20873	49,6	8,3	T1c	NX	MX	7	No	
PC_4786	55,4	10,2	T1c	NX	M0	7	Yes	1,9
PC_4906	58,3	4	T2	NX	MX	6	No	
PC_4980	59,7	3,5	T1c	NX	MX	6	No	
PC_17163	56,8	19,8	T2	NX	M0	7	Yes	1,1

Supplementary Table 2. Description of CRPC samples used in the study.

Sample ID	Sample type	Hormonal treatment
CRPC_278	CRPC	orchiectomy
CRPC_305	CRPC	bicalutamide+orchiectomy
CRPC_348	CRPC	orchiectomy
CRPC_435	CRPC	LHRH
CRPC_489	CRPC	LHRH
CRPC_530	CRPC	LHRH+bicalutamide
CRPC_531	CRPC	orchiectomy
CRPC_539	CRPC	LHRH analog
CRPC_541	CRPC	estrogen
CRPC_543	CRPC	orchiectomy
CRPC_697	CRPC	orchiectomy

Supplementary Table 3a. Numbers of common samples between the proteomics and other analyses

	BPH	PC	CRPC	all
proteomics	10	17	11	38
mRNA (RNAseq)	6	17	11	34
CNA	0	15	8	23
methylation	6	16	9	31
miRNA	0	17	11	28

Supplementary Table 3b. Sample-wise overlap between the proteomics and other analyses

0 = not available
1 = available

	BPH_335	BPH_337	BPH_456	BPH_656	BPH_664	BPH_671	BPH_677	BPH_689	BPH_718	BPH_719
Protein	1	1	1	1	1	1	1	1	1	1
mrna	0	1	1	1	0	1	1	1	0	0
CNA	0	0	0	0	0	0	0	0	0	0
methylation	0	1	1	1	0	1	1	1	0	0
miRNA	0	0	0	0	0	0	0	0	0	0

	PC_10286	PC_12517	PC_14670	PC_15194	PC_15420	PC_15760	PC_17163	PC_17447	PC_18307	PC_20873
Protein	1	1	1	1	1	1	1	1	1	1
mrna	1	1	1	1	1	1	1	1	1	1
CNA	1	1	0	1	1	1	1	1	1	1
methylation	1	1	1	1	1	1	1	1	1	1
miRNA	1	1	1	1	1	1	1	1	1	1

	PC_470	PC_4786	PC_4906	PC_4980	PC_5934	PC_8131	PC_9324
Protein	1	1	1	1	1	1	1
mrna	1	1	1	1	1	1	1
CNA	1	1	1	1	1	1	0
methylation	1	1	0	1	1	1	1
miRNA	1	1	1	1	1	1	1

	CRPC_278	CRPC_305	CRPC_348	CRPC_435	CRPC_489	CRPC_530	CRPC_531	CRPC_539	CRPC_541	CRPC_543	CRPC_697
Protein	1	1	1	1	1	1	1	1	1	1	1
mrna	1	1	1	1	1	1	1	1	1	1	1
CNA	1	0	0	1	1	1	1	0	1	1	1
methylation	1	0	1	1	1	1	1	0	1	1	1
miRNA	1	1	1	1	1	1	1	1	1	1	1

Supplemetary Table 4. Validated exonic point mutations in the samples of the study.

A. Germline mutations

	impact_score_mr	impact_score_pr	CHROM	POSITION	REF	ALT	EXONIC_FUNCTION	samples
ETFDH:159620229	-4,273888816	-0,54273593	chr4	159620229	GG	-	frameshift deletion	['PC_10286']
COPE:19016431	-4,004980013	-0,037039381	chr19	19016431	T	C	nonsynonymous SNV	['PC_5934']
IARS2:220307849	-2,421725442	-1,190784815	chr1	220307849	A	C	nonsynonymous SNV	['PC_8131']
RGN:46951576	-2,305673119	-1,681312431	chrX	46951576	G	A	nonsynonymous SNV	['CRPC_278']
MTHFD1L:1513582	-1,954600174	-2,822050963	chr6	151358205	C	G	nonsynonymous SNV	['PC_15420']
ASNS:97484750	-1,871877712	-1,630845781	chr7	97484750	T	C	nonsynonymous SNV	['PC_10286']
RPTOR:78935195	-1,829344006	0,803092254	chr17	78935195	C	T	nonsynonymous SNV	['PC_15760']
MPP6:24720066	-1,803920348	-1,788661812	chr7	24720066	C	G	nonsynonymous SNV	['PC_15760']
ALDH9A1:1656486	-1,741794931	-6,414159836	chr1	165648699	T	C	nonsynonymous SNV	['PC_20873']
EIF5:103804756	-1,63902985	0,38031895	chr14	103804756	A	G	nonsynonymous SNV	['PC_18307']
CPE:166388880	-1,574507982	-0,403235961	chr4	166388880	C	T	nonsynonymous SNV	['PC_15194']
PEPD:33892648	-1,451834049	2,152853751	chr19	33892648	C	T	nonsynonymous SNV	['PC_12517']
RANBP2:10938179	-1,425637825	-0,02828696	chr2	109381791	T	C	nonsynonymous SNV	['PC_470']
CHAF1B:37787599	-1,250748131	-0,547676391	chr21	37787599	A	G	nonsynonymous SNV	['PC_5934']
DPM3:155112667	-1,215943482	-5,065267165	chr1	155112667	G	A	nonsynonymous SNV	['PC_4980']
LMNB1:12614593(	-0,850252728	-3,593321509	chr5	126145930	G	A	nonsynonymous SNV	['PC_15194']
SPAG9:49074053	-0,824631259	-1,18179288	chr17	49074053	G	C	nonsynonymous SNV	['PC_18307']
CTSH:79214537	-0,691312942	-2,412403692	chr15	79214537	T	G	nonsynonymous SNV	['PC_4980']
UBQLN2:56591304	-0,663752156	0,540432242	chrX	56591304	G	A	nonsynonymous SNV	['PC_470']
UBQLN2:56591220	-0,663752156	0,540432242	chrX	56591220	C	T	nonsynonymous SNV	['PC_470']
VPS4B:61066514	-0,564056986	-2,280248035	chr18	61066514	G	A	nonsynonymous SNV	['PC_12517']
HSP90B1:1043411	-0,535409613	0,17095656	chr12	104341190	A	T	nonsynonymous SNV	['PC_20873', 'PC_8131']
CTSZ:57581475	-0,484079558	-1,801491478	chr20	57581475	T	C	nonsynonymous SNV	['PC_12517']
OSGEP:20915587	-0,475402189	0,098074718	chr14	20915587	G	A	nonsynonymous SNV	['PC_5934']
ABCD1:153002679	-0,346700329	-1,42792387	chrX	153002679	G	A	nonsynonymous SNV	['PC_14670']
RPL14:40499417	-0,335296223	-0,98264535	chr3	40499417	T	C	nonsynonymous SNV	['PC_20873']
TARS:33461278	-0,284917868	-1,704888314	chr5	33461278	A	G	nonsynonymous SNV	['PC_12517']
SQRDL:45968461	-0,13960676	-1,950509575	chr15	45968461	G	T	nonsynonymous SNV	['PC_12517']
TMEM70:7489365	-0,116885593	-2,822330142	chr8	74893650	AC	-	frameshift deletion	['PC_20873']
COG1:71189276	-0,074543771	1,237121775	chr17	71189276	A	G	nonsynonymous SNV	['PC_4906']
UBR4:19484329	0,019359305	-0,938361369	chr1	19484329	C	T	nonsynonymous SNV	['CRPC_435']
NUBP2:1837966	0,042168735	0,396842622	chr16	1837966	C	T	nonsynonymous SNV	['PC_4906']
TNS1:218678425	0,051673179	1,123283793	chr2	218678425	A	T	nonsynonymous SNV	['PC_17163']
ME1:83963448	0,172284302	1,406340813	chr6	83963448	C	A	nonsynonymous SNV	['PC_20873']
CPT2:53662764	0,238348996	-1,306425787	chr1	53662764	C	A	nonsynonymous SNV	['PC_5934']
PLEC:144996110	0,270304018	-3,222110154	chr8	144996110	G	A	nonsynonymous SNV	['PC_12517']
PRRC2A:31599160	0,293474963	1,72655447	chr6	31599160	C	T	nonsynonymous SNV	['PC_5934']
GGT1:25011062	0,353347597	-1,210108359	chr22	25011062	C	G	nonsynonymous SNV	['PC_12517']
PODXL2:12738739	0,367683536	-0,693133729	chr3	127387398	G	A	nonsynonymous SNV	['PC_470']
PRPF40A:1535191	0,429509473	-2,145094997	chr2	153519194	T	C	nonsynonymous SNV	['PC_17163']
C16orf58:3150494	0,501838512	-0,304870968	chr16	31504947	T	C	nonsynonymous SNV	['PC_14670']
AP2A1:50304686	0,536094118	0,447112847	chr19	50304686	T	A	nonsynonymous SNV	['PC_10286']
SPR:73114562	0,576270434	-0,663548577	chr2	73114562	A	C	nonsynonymous SNV	['PC_10286']
DPT:168683499	0,679950556	-0,011376111	chr1	168683499	A	G	nonsynonymous SNV	['PC_4980']
POLR2A:7406475	0,796541938	0,574051297	chr17	7406475	G	A	nonsynonymous SNV	['PC_10286']
FLNA:153581670	0,825557059	3,535726491	chrX	153581670	G	A	nonsynonymous SNV	['PC_15420']
FLNA:153581200	0,825557059	3,535726491	chrX	153581200	A	T	nonsynonymous SNV	['PC_15420']
KRT17:39775857	0,905159486	-4,85332262	chr17	39775857	T	C	nonsynonymous SNV	['PC_4786']
DIAPH1:14095155	0,912825406	2,375665048	chr5	140951550	A	C	nonsynonymous SNV	['CRPC_489']
PLEC:145004655	0,99934132	1,682492752	chr8	145004655	G	A	nonsynonymous SNV	['PC_17163']
ACE:61574343	1,034011502	-0,18074709	chr17	61574343	T	G	nonsynonymous SNV	['PC_12517']
COL6A2:47552167	1,149600811	-0,431916679	chr21	47552167	G	A	nonsynonymous SNV	['PC_20873']
PABPC1:10171896	1,247821824	-0,451516425	chr8	101718964	C	T	nonsynonymous SNV	['PC_20873']
MYH9:36722663	1,268606229	4,509180978	chr22	36722663	T	C	nonsynonymous SNV	['PC_20873']
CA3:86351166	1,305788345	2,487943429	chr8	86351166	C	T	nonsynonymous SNV	['PC_8131']
PALM:746714	1,343015829	-1,369622987	chr19	746714	C	T	nonsynonymous SNV	['PC_15194']
FLII:18160308	1,370465668	-2,472867819	chr17	18160308	T	G	nonsynonymous SNV	['PC_4906']
UBE2O:74392608	1,513313035	-2,786718769	chr17	74392608	T	C	nonsynonymous SNV	['PC_4786']
SPINT2:38780888	1,669515196	-0,863638812	chr19	38780888	G	A	nonsynonymous SNV	['PC_15420']
GOLGA2:1310249	1,842791732	-0,960268864	chr9	131024970	C	G	nonsynonymous SNV	['PC_8131']
UBAP2L:15422177	2,109949591	0,651944432	chr1	154221776	G	A	nonsynonymous SNV	['PC_15194']
ELN:73477965	3,527676502	2,99193953	chr7	73477965	G	A	nonsynonymous SNV	['CRPC_697']

Supplemetary Table 4. Validated exonic point mutations in the samples of the study.

B. Somatic mutations

	impact_score_mr	impact_score_pr	CHROM	POSITION	REF	ALT	EXONIC_FUNCTION	samples
FOXA1:38061231	-18,71217922	-1,300208368	chr14	38061231	A	T	nonsynonymous SNV	['PC_10286']
GIGYF2:23365192(	-3,198048213	-0,867832628	chr2	233651920	G	A	nonsynonymous SNV	['PC_15194']
FOXA1:38061862	-3,130111294	0,027570643	chr14	38061862	A	T	nonsynonymous SNV	['CRPC_531']
CHD4:6700867	-2,995076236	2,772231077	chr12	6700867	A	G	nonsynonymous SNV	['CRPC_489']
CTNNB1:41266097	-1,919718291	1,376822462	chr3	41266097	G	T	nonsynonymous SNV	['CRPC_278']
ACO2:41923373	-1,597392883	-0,882920942	chr22	41923373	C	T	nonsynonymous SNV	['PC_15420']
DNAJC8:28527842	-0,736821923	2,011982114	chr1	28527842	G	A	stopgain SNV	['PC_5934']
RPL8:146015286	-0,274389122	0,831359775	chr8	146015286	C	T	nonsynonymous SNV	['PC_8131']
OSBPL8:76769065	-0,062942687	1,56070343	chr12	76769065	T	A	nonsynonymous SNV	['PC_4786']
BDH1:197239092	-0,044832453	-1,289222392	chr3	197239092	C	A	nonsynonymous SNV	['CRPC_435']
SSRP1:57099223	0,088695461	0,512964912	chr11	57099223	C	T	nonsynonymous SNV	['PC_4786']
SELENBP1:151339	0,098027497	-0,357692181	chr1	151339341	T	C	nonsynonymous SNV	['PC_17163']
KIDINS220:889032	0,799550215	0,123625761	chr2	8890321	G	T	nonsynonymous SNV	['CRPC_539']
HAT1:172848181	1,061823611	-0,61110626	chr2	172848181	G	A	nonsynonymous SNV	['CRPC_435']
HNRNPR:2364507(	1,707664207	1,300541598	chr1	23645070	C	T	nonsynonymous SNV	['PC_470']
AIM1:106968097	1,878314166	0,811693291	chr6	106968097	C	T	nonsynonymous SNV	['PC_15194']
SRP68:74036543	2,120077794	-0,730976967	chr17	74036543	G	C	nonsynonymous SNV	['PC_470']
EIF3I:32692064	2,168386633	0,231372746	chr1	32692064	G	C	nonsynonymous SNV	['CRPC_539']
COX5B:98262619	3,507438371	0,529914215	chr2	98262619	G	A	nonsynonymous SNV	['CRPC_539']
TARDBP:11082217	3,665980066	-2,820575209	chr1	11082217	A	G	nonsynonymous SNV	['CRPC_489']

Supplementary Table 5. Correlation of protein expression with mutation load.

Point mutations
(correlation >0.5 or <-0.5)

gene	correlation
PLIN4	-0,5974
PAIP2	-0,55501
NOL3	-0,5276
ABCF2	0,514033
DAP3	0,55614
C8orf82	0,560096
UQCR10	0,569421
DARS2	0,575638
PHB	0,582986
CAD	0,594007
PRDX3	0,645156

Copy number alterations
(correlation >0.75 or <=-0.75)

gene	correlation
COPG1	-0,88538
NANS	-0,86462
IQGAP2	-0,82016
TAGLN	-0,79283
GMDS	-0,77372
STXBP3	-0,77273
UCHL3	-0,76581
SEC14L2	-0,76383
ANXA5	-0,75958
MAN2B2	-0,75
PFDN6	0,750988
TOMM40	0,753953
SHMT2	0,753953
SNRPF	0,768775
LASP1	0,768775
VDAC2	0,774704
VDAC1	0,796443

Rearrangements
(correlation >0.7 or <-0.7)

gene	correlation
TLN2	-0,79075
TLN1	-0,75628
EIF3D	-0,74013
PSMD12	-0,72748
MDH1	-0,72704
PFKP	-0,72093
EHD2	-0,70086
ATP5D	0,702165
RAN	0,704783
TIMM8B	0,715693
TMSB4X	0,72704
RPL27A	0,729222
C7orf55	0,729658
CARKD	0,741441
COX5B	0,747114
SPARC	0,767188
SLTM	0,767188

Supplementary Table 6. miRNAs and their negatively correlating (<-0.50) targets as identified based on differential mRNA expression

miRNA	correlating mRNA (targets correlating also at the protein level marked in red colour)

differentially expressed miRNAs in italics

miRNAs identified in several analyses highlighted with colour.

correlated in PC

hsa-miR-1193	['GINS4', 'SYNPO']
hsa-miR-181c-5p	['NDRG2', 'ACTC1', 'HMGB2']
hsa-miR-23a-3p	['DDX39B', 'MRGPRF', 'PAGE4']
hsa-miR-3150a-5p	['LPCAT1']
hsa-miR-3661	['AR', 'PC', 'NFIX']
hsa-miR-382-5p	['KIAA1210', 'PARVA', 'MAN2B2']
hsa-miR-3914	['LMNB1', 'AR', 'NFIX']
hsa-miR-4664-3p	['SEC14L2']
hsa-miR-491-3p	['XPNPEP3', 'GRB10']
hsa-miR-615-5p	['CSRP1', 'PPP1R12B', 'GPX3', 'PGM5', 'DES', 'LSAMP', 'GNAO1', 'ARRB1']
hsa-miR-92a-2-5p	['TMEM35', 'LSAMP', 'CNN1', 'SLC25A4', 'GLIPR2', 'ACTC1', 'LDB3', 'PEBP4', 'GNAO1', 'MYLK', 'ARRB1']

correlated in CRPC

hsa-miR-106b-5p	['FXYD3', 'CFL2', 'PPP1R12B', 'PTGIS', 'PDLIM4', 'PARVA', 'MYADM', 'SYNM', 'PGM5', 'TGFB1I1', 'PTRF', 'TNKS1BP1', 'PRELP', 'CNN1', 'MYO1C', 'ALOX15B', 'TNS1']
hsa-miR-125a-3p	['ABAT', 'HM13', 'EIF5', 'REEP6', 'ARRB1']
hsa-miR-182-5p	['CACNA2D1', 'SYNPO', 'SH3BGRL', 'MYO1C', 'LDB3', 'LMOD1', 'TMPRSS2', 'ALDH1A2', 'SLC4A1']
hsa-miR-194-3p	['HM13', 'COMT', 'LPCAT1', 'SMC4']
hsa-miR-205-5p	['GTPBP10', 'XPNPEP3', 'AR', 'PNN']
hsa-miR-23a-3p	['OGT', 'DDX39B', 'ACADSB', 'XPNPEP3', 'AR', 'PNN', 'NFIX']
hsa-miR-3065-3p	['CPE', 'HEXA', 'LMOD1', 'TNS1', 'PCCA']
hsa-miR-30c-1-3p	['ABAT', 'HSPB6', 'MYADM', 'MYH11', 'SYNPO', 'PLIN4', 'TNS1', 'ASNA1', 'GNAO1']
hsa-miR-3162-3p	['CSRP1', 'TMEM35', 'PTGIS', 'SLC25A35', 'PRELP', 'PDLIM7', 'SORBS1', 'GNAO1']
hsa-miR-3619-5p	['SLC9A3R2', 'PPP1R12B', 'GMPR', 'C16orf58', 'VAMP2', 'ACPP', 'GLIPR2', 'BAIAP2', 'MYO1C', 'LDB3', 'PEBP4', 'ARRB1']
hsa-miR-3929	['SLC9A3R2', 'OGT', 'ACADSB', 'MTDH', 'XPNPEP3', 'AR', 'BAIAP2', 'NFIX']
hsa-miR-4645-5p	['HM13', 'ACADSB', 'XPNPEP3', 'PDCD11', 'SSBP1']
hsa-miR-4649-3p	['FANCD2', 'HM13', 'XPNPEP3', 'GNMT', 'SSBP1']
hsa-miR-4748	['SLC9A3R2', 'PPP1R12B', 'GMPR', 'C16orf58', 'VAMP2', 'GLIPR2', 'LDB3', 'PLIN4', 'PEBP4']
hsa-miR-551b-5p	['GINS4', 'LPCAT1', 'TMPO', 'ZNF185', 'TOP2A', 'MTDH', 'WDHD1', 'CBX3', 'HMGB2']
hsa-miR-93-5p	['FXYD3', 'CFL2', 'PTGIS', 'PDLIM4', 'PARVA', 'MYADM', 'TGFB1I1', 'PTRF', 'TNKS1BP1', 'PRELP', 'CNN1', 'ALOX15B', 'TNS1']

correlated in both PC and CRPC

hsa-miR-101-3p	['GINS4', 'EIF5', 'TMEM65', 'STMN1', 'VCAN']
hsa-miR-23a-3p	['FANCD2', 'TMPO', 'OGT', 'DDX39B', 'EIF5', 'TOP2A', 'MTDH', 'XPNPEP3', 'PNN', 'HMGB2', 'SSBP1']
hsa-miR-23b-3p	['LMNB1', 'CPOX', 'MCM4', 'FANCD2', 'TMPO', 'DDX39B', 'EIF5', 'TOP2A', 'MTDH', 'XPNPEP3', 'PNN', 'NCAPG', 'HMGB2']
hsa-miR-424-3p	['NFIX']
hsa-miR-454-3p	['KIAA1210', 'PARVA', 'MAP2K1', 'MYADM', 'TNS1', 'GNAO1']

Supplementary Table 7. miRNAs and their negatively correlating (<-0.50) targets as identified based on differential protein expression

miRNA	correlating protein
differentially expressed miRNAs in italics	
miRNAs identified in several analyses highlighted with colour.	
**correlated in PC**	
hsa-miR-1255a	['AOC3', 'GNAO1', 'PARVA', 'CNP', 'ATP2A2']
hsa-miR-1289	['CDV3', 'PARVA', 'ATP2A2']
hsa-miR-2355-3p	['CD109', 'SEC24C', 'ERLIN2']
hsa-miR-23b-5p	['HDGF', 'CALU']
hsa-miR-25-5p	['ARHGAP1', 'ATL3', 'VWA1', 'LIMS2']
hsa-miR-3065-5p	['SRSF1', 'NUDT21', 'MATR3', 'TCOF1', 'SRSF7', 'MIA3']
hsa-miR-3180-3p	['GNAO1', 'ATP2A2', 'TGFB1I1']
hsa-miR-3200-3p	['PLG', 'SEC14L2', 'VCL']
hsa-miR-4485	['BCLAF1', 'MMAB']
hsa-miR-561-5p	['SEC24A', 'SDC1', 'PARVA', 'ATP2A2', 'NXN', 'FERMT2', 'PRKAR2A', 'COL1A1']
hsa-miR-5688	['DERL1', 'VDAC2', 'NUDCD1']
hsa-miR-618	['ACPP', 'RPL23A']
hsa-miR-96-5p	['LDB3', 'LPP', 'VCL', 'LAMC1', 'PGM5', 'PALLD']
**correlated in CRPC**	
hsa-miR-106b-5p	['FOXA1', 'ARHGAP1', 'PARVA', 'LASP1', 'TNS1', 'PGM5', 'PALLD']
hsa-miR-1262	['PPP2R5D', 'PAICS', 'MXRA7', 'CCT6A', 'COPA']
hsa-miR-1285-3p	['ACADSB', 'ATP2B4', 'XPNPEP3', 'OXSR1', 'B4GALT5', 'RDH11', 'ALDH6A1']
hsa-miR-136-5p	['AGL', 'LDB3', 'TFRC', 'PGM3', 'DCP1A', 'AP1G1']
hsa-miR-197-5p	['ACADSB', 'PFDN2', 'ADH1B', 'RAC2', 'NCSTN']
hsa-miR-222-3p	['SNRPD3', 'MTDH', 'SUB1', 'SRSF1', 'TFRC', 'FUBP1', 'ATP1B1', 'CAPRIN1', 'STMN1']
hsa-miR-22-3p	['KHSRP', 'RAB3B', 'PFDN6', 'B4GALT5', 'GUK1', 'SERBP1', 'MDH2']
hsa-miR-29a-3p	['HDGF', 'LAMTOR1', 'SUB1', 'FUBP1', 'TCOF1', 'ATP1B1', 'CAPRIN1']
hsa-miR-3130-5p	['BROX', 'PARVA', 'MYLK', 'VCL', 'AP1G1', 'SERPINA7']
hsa-miR-3135a	['XPNPEP3', 'MATR3', 'SMN1', 'CPE']
hsa-miR-3150a-5p	['GMDS', 'SNTB2', 'PPP2R4']
hsa-miR-3155a	['HECTD3', 'CNBP', 'FABP3', 'MXRA7', 'TOM1L2', 'TTC38', 'TOLLIP']
hsa-miR-326	['OGDH', 'PPP2R5D', 'ATP2B4', 'OXSR1', 'ATP2A2', 'ERLIN2']
hsa-miR-3921	['SORBS1', 'ARHGAP1', 'ACTN1', 'COMT', 'PARVA', 'ILK', 'PGM3', 'PYGB', 'MSN', 'EHD2', 'PRKAR2A']
hsa-miR-432-3p	['UAP1', 'SDC1', 'CD276', 'CD34', 'KIF2A']
hsa-miR-4454	['ARHGAP1', 'PYGB', 'MSN']
hsa-miR-450a-5p	['CCT6A', 'SNTB2']
hsa-miR-4732-3p	['EML2', 'CDV3', 'MXRA7', 'NENF', 'MAP4', 'ZNF512']
hsa-miR-4772-3p	['ATP6V1C1', 'XPNPEP3', 'PDCD6IP', 'SUB1', 'GNA11', 'MATR3', 'TOR1AIP1']
hsa-miR-4783-3p	['ATP6V1C1', 'XPNPEP3', 'GNA11', 'TOM1L2']
hsa-miR-485-5p	['MPI', 'CNBP', 'B4GALT5', 'MXRA7', 'TOM1L2', 'TOLLIP']
hsa-miR-490-5p	['BID', 'LDB3', 'GNAO1', 'PARVA', 'FERMT2', 'AP1G1']
hsa-miR-493-5p	['AGL', 'PPP2R5D', 'ATL3', 'GBE1', 'ATP2A2', 'DNM1L', 'OLA1']
hsa-miR-629-3p	['VASP', 'GNAO1', 'SEC14L2', 'MXRA7']
hsa-miR-7-5p	['RPP30', 'GNAO1', 'PSME3', 'ANXA11']
hsa-miR-873-3p	['CNBP', 'CNP', 'B4GALT5', 'MXRA7', 'TTC38']
**correlated in both PC and CRPC**	
hsa-miR-130a-3p	['OGDH', 'HADHA', 'TOM1L2', 'CAB39L']
hsa-miR-132-3p	['HBD', 'VDAC2', 'FUBP1', 'FUS', 'CAPRIN1']
hsa-miR-200c-3p	['SEC24A', 'PARVA', 'SACM1L', 'ATP2A2', 'PSMF1', 'DSTN', 'FERMT2', 'NUP153', 'MSN', 'IQGAP1']
hsa-miR-21-5p	['LPP', 'SNTB2']
hsa-miR-3150a-5p	['GMDS']
hsa-miR-3175	['LDB3', 'GNAO1', 'CSRP1', 'TNS1', 'DPYSL3', 'TLN1', 'EHD2']
hsa-miR-3921	['ARHGAP1', 'TGFB1I1', 'PRKAR2A']
hsa-miR-5706	['ENTPD1', 'KIF2A']
hsa-miR-625-3p	['SEC24A', 'SNX2', 'PDLIM5', 'COPA', 'PAFAH1B2']
hsa-miR-92a-1-5p	['FOXA1', 'DPT', 'RCN3', 'CD82']
hsa-miR-93-5p	['GSTM3', 'VASP', 'ALDH1A3', 'LDB3', 'ARHGAP1', 'PARVA', 'ATL3', 'SNX2', 'TNS1', 'DNM1L', 'LPP', 'GNA11', 'CMPK1', 'TGFB1I1', 'CLTC', 'FAM129A', 'STARD10', 'SNTB2', 'RNH1', 'PGM5', 'PALLD', 'PRKAR1A', 'PRKAR2A']
hsa-miR-99b-3p	['SEC24A', 'ATP2B4', 'FAM129A']

Supplementary Table 8. Genes regulated by differentially expressed miRNAs according to both mRNA and protein expression

ACO2
ACPP
ACSL3
AGL
AK4
ALAD
ALDH1A2
ANXA11
AP3S2
ARHGAP1
ATP2A2
ATP2B4
ATP6V1C1
B4GALT5
BCAS1
CACYBP
CANX
CBR1
CCT5
CCT6A
CD59
CFL2
CHERP
CLIC4
CNN1
COL1A2
COL3A1
COMT
CORO1B
COX20
CPD
CRYM
CTSB
CTTN
DBI
DKC1
DPYSL3
DUSP3
EML4
ENDOD1
FAM129A
FERMT2
FNBP1
GLG1
GNAO1
GNE
GOLIM4
GPD1L
GSK3B
HMGB2
HNMT
HSPB6
ICAM1
KIAA1468
L2HGDH
LAMP2
LDB3
LMNB1
LMOD1
LPP
LRPPRC
LRRC59
MAN2B2
MAT2A
MTDH
MYLK
NCLN
NDUFA10
NLN
OXSR1
PARVA
PFKP
PGM5
PKP1
PLS3
PNN
PPP1CB
PPP1R12B
PYCRL
PYGB
RDH11
RSU1
SAR1B
SCARB2
SEC14L2
SEC24C
SH2D4A
SNRPE
SNRPF
SNTB2
SNX1
SORBS1
SPARC
SRSF1
SSBP1
STARD10
STK39
STMN1
SURF4
SYNPO2
TBXAS1
TES
TGFB1I1
TLN1
TMEM35
TMEM65
TMPO
TNS1
TOM1L2
TPM1
TRA2B
TRIM25
TTC38
TTLL12
UFL1
VASP
VCAN
VCL
VKORC1L1
XPNPEP3
XPO1
ZNF185

Supplementary Table 9. Differentially expressed genes regulated by differentially expressed miRNAs according to mRNA expression data

ABAT
ACADSB
ACPP
AIDA
ALDH1A2
ALOX15B
ANPEP
AR
ARG2
ARL6IP5
ARRB1
BAIAP2
C16orf58
CACNA2D1
CAP2
CBX3
CCT6A
CDC42EP4
CFL2
CHORDC1
CNN1
COG3
COMT
CPOX
CRYM
DDX39B
DNAJC7
EIF5
ENDOD1
FANCD2
FXYD3
GINS4
GLIPR2
GNAO1
GOLM1
GRB10
GTPBP10
HEXA
HMGB2
HNRNPA1
HSPB6
HSPD1

ITGA5
KIAA1210
KRT17
LDB3
LMNB1
LMOD1
LPCAT1
LSAMP
MAN2B2
MAP2K1
MCM4
MCM7
MME
MPDU1
MRPL15
MTDH
MTHFD1L
MYADM
MYH11
MYLK
NCAPG
NCAPH
NDRG2
NEBL
NFIX
OGT
ORM1
PARVA
PBK
PDLIM4
PEBP4
PGM5
PKP1
PNN
PPP1R12B
PRELP
PRSS8
PTGIS
PTRF
PYGB
REEP6
RRS1

SEC14L2
SLC44A4
SLC4A1
SLC9A3R2
SMC4
SNRPF
SORBS1
SSBP1
STARD10
STMN1
SYNM
SYNPO
TACSTD2
TGFB1I1
TLN1
TMEM35
TMEM65
TMPO
TMPRSS2
TNS1
TOP2A
TRIM25
UAP1L1
UBR5
VCAN
VDAC3
WDHD1
XPNPEP3
XPO1
YWHAZ
ZNF185

Supplementary Table 10. Differentially expressed genes regulated by differentially expressed miRNAs according to protein expression data

ACO2
ACPP
ACSL3
ACTN1
ADH1B
ADH5
AGL
ALDH1A3
ALDH6A1
ALYREF
ANP32B
ANXA11
ANXA4
ANXA6
AP1G1
ARHGAP1
ATL3
ATP1B1
ATP2A2
ATP2B4
ATP6V1C1
AZGP1
B4GALT5
BCLAF1
BID
BST2
BZW1
CAB39L
CALU
CAPRIN1
CBR1
CCDC124
CCT6A
CD109
CD276
CD82
CFHR1
CKB
CLNS1A
CLTC
CLU
CMPK1

CNBP
CNP
CNPY2
CNPY4
COL1A1
COL1A2
COL3A1
COMT
COMTD1
COPA
COPG1
CORO1B
COX5A
CRYM
CSRP1
CYFIP1
DCP1A
DDX19A
DERL1
DNM1L
DPP4
DPYSL3
DSTN
DUSP3
EHD2
EIF5A
EMILIN1
ENTPD1
ERH
FABP3
FAM129A
FAM98B
FERMT2
FKBP11
FLNB
FOXA1
FUBP1
FUS
GGT1
GNA11
GNAO1
GSTM3

HADHA
HBB
HBD
HDLBP
HID1
HLA-DRB1
HNMT
HNRNPU
HSPA1B
IFITM1
IQGAP1
IQGAP2
KHSRP
KLK11
LACTB2
LAMTOR1
LASP1
LDB3
LPP
MAN2B2
MAP4
MATR3
MDH2
MIA3
MMAB
MPI
MRPL12
MSN
MTDH
MXRA7
MYL12A
MYLK
NACA
NANS
NDUFA10
NHP2L1
NIPSNAP3A
NPM1
NUDT21
NXN
OLA1
OTUB1

OXSR1
PACSIN2
PAFAH1B2
PAICS
PALLD
PARVA
PCBP2
PDCD6IP
PDLIM5
PFDN6
PGM3
PGM5
PLG
PPIL1
PPP2R5D
PPP3CA
PPP6R3
PRAF2
PRKACA
PRKAR1A
PRKAR2A
PRKCSH
PSMD10
PSME3
PSMF1
PTMA
PTMS
PYGB
RAB7A
RBMX
RCN3
RDH11
RNH1
RPL23A
RPP30
SACM1L
SAFB
SAR1B
SEC14L2
SEC24A
SEC24C
SERBP1
SERPINA1
SH3BGRL3
SMN1

SNRPD3
SNRPE
SNRPF
SNTB2
SNX2
SORBS1
SPARC
SRSF1
SRSF2
SRSF7
SSBP1
STARD10
STIP1
STMN1
STXBP3
SUB1
SURF4
TAGLN
TARDBP
TCEA1
TFRC
TGFB1I1
THY1
TIMM9
TLN1
TM9SF2
TMPO
TNS1
TOM1L2
TOMM20
TOMM22
TOMM40
TPD52L2
TTC38
TXNL1
UCHL3
VASP
VCAN
VCL
VDAC1
VDAC2
WDR1
VIM
VTA1
XPNPEP3

XRCC5
ZNF512

Supplementary Table 11. Most significantly (p<0.05) altered pathways based on proteomics data by Ingenuity Pathway Analysis.

Common pathways in bold

PC vs. BPH		CRPC vs. PC	
IPA pathway	-log(p-value)	IPA pathway	-log(p-value)
EIF2 Signaling	13,7	ILK Signaling	7,91E+00
Regulation of eIF4 and p70S6K Signaling	9,11	Glycogen Degradation II	6,04E+00
FXR/RXR Activation	8,06	Glycogen Degradation III	5,61E+00
Caveolar-mediated Endocytosis Signaling	7,93	GDP-glucose Biosynthesis	5,40E+00
LXR/RXR Activation	7,61	Noradrenaline and Adrenaline Degradation	5,25E+00
Isoleucine Degradation I	6,86	Glucose and Glucose-1-phosphate Degradation	5,11E+00
Colanic Acid Building Blocks Biosynthesis	6,86	Dopamine Receptor Signaling	4,84E+00
GDP-mannose Biosynthesis	6,59	TCA Cycle II (Eukaryotic)	4,25E+00
Clathrin-mediated Endocytosis Signaling	6,48	Actin Cytoskeleton Signaling	4,24E+00
Fatty Acid Î²-oxidation I	6,11	Tight Junction Signaling	4,14E+00
Atherosclerosis Signaling	6,01	Mitochondrial Dysfunction	4,04E+00
Valine Degradation I	5,95	Caveolar-mediated Endocytosis Signaling	3,46E+00
Acute Phase Response Signaling	5,92	Ethanol Degradation II	3,35E+00
mTOR Signaling	5,81	UDP-N-acetyl-D-galactosamine Biosynthesis II	3,31E+00
Actin Cytoskeleton Signaling	5,35	NRF2-mediated Oxidative Stress Response	3,24E+00
Leucine Degradation I	5,31	Calcium Transport I	3,16E+00
Telomere Extension by Telomerase	5,25	Netrin Signaling	3,13E+00
TCA Cycle II (Eukaryotic)	5,12	Tryptophan Degradation X (Mammalian, via Tryptamin	3,10E+00
Ketogenesis	5,02	Neuroprotective Role of THOP1 in Alzheimer's Disease	3,08E+00
Unfolded protein response	4,94	D-glucuronate Degradation I	2,99E+00
Signaling by Rho Family GTPases	4,79	Virus Entry via Endocytic Pathways	2,86E+00
Agrin Interactions at Neuromuscular Junction	4,73	Serotonin Degradation	2,81E+00
Integrin Signaling	4,58	Remodeling of Epithelial Adherens Junctions	2,78E+00
Complement System	4,57	Dopamine Degradation	2,77E+00
Glutaryl-CoA Degradation	4,55	Integrin Signaling	2,75E+00
D-glucuronate Degradation I	4,41	Intrinsic Prothrombin Activation Pathway	2,71E+00
Intrinsic Prothrombin Activation Pathway	4,4	Isoleucine Degradation I	2,70E+00
Hepatic Fibrosis / Hepatic Stellate Cell Activation	4,28	CDK5 Signaling	2,60E+00
Regulation of Actin-based Motility by Rho	4,25	CMP-N-acetylneuraminate Biosynthesis I (Eukaryotes)	2,48E+00
RhoA Signaling	4,18	Cardiac Î²-adrenergic Signaling	2,46E+00
Remodeling of Epithelial Adherens Junctions	4,04	Histamine Degradation	2,45E+00
Epithelial Adherens Junction Signaling	3,88	Valine Degradation I	2,37E+00
NRF2-mediated Oxidative Stress Response	3,86	UDP-N-acetyl-D-glucosamine Biosynthesis II	2,31E+00
Ketolysis	3,84	Granzyme A Signaling	2,24E+00
Leukocyte Extravasation Signaling	3,84	Regulation of eIF4 and p70S6K Signaling	2,21E+00
Glycolysis I	3,82	Protein Kinase A Signaling	2,21E+00
Gluconeogenesis I	3,82	Putrescine Degradation III	2,18E+00
ILK Signaling	3,72	Acetyl-CoA Biosynthesis I (Pyruvate Dehydrogenase Co	2,17E+00
RhoGDI Signaling	3,57	IL-1 Signaling	2,15E+00
Mechanisms of Viral Exit from Host Cells	3,39	Clathrin-mediated Endocytosis Signaling	2,10E+00
Granzyme A Signaling	3,35	LPS/IL-1 Mediated Inhibition of RXR Function	2,07E+00
Tryptophan Degradation III (Eukaryotic)	3,25	Dopamine-DARPP32 Feedback in cAMP Signaling	1,97E+00
Mevalonate Pathway I	3,14	Ketolysis	1,94E+00
Virus Entry via Endocytic Pathways	3,07	Signaling by Rho Family GTPases	1,90E+00
Coagulation System	2,99	Ketogenesis	1,85E+00
Acetyl-CoA Biosynthesis I (Pyruvate Dehydrogenase Co	2,91	Amyloid Processing	1,83E+00
RAN Signaling	2,77	Aryl Hydrocarbon Receptor Signaling	1,80E+00
tRNA Charging	2,73	Glutathione-mediated Detoxification	1,79E+00
Aldosterone Signaling in Epithelial Cells	2,7	Ceramide Signaling	1,78E+00
Superpathway of Geranylgeranyldiphosphate Biosynth	2,66	Androgen Signaling	1,75E+00
Superpathway of Cholesterol Biosynthesis	2,65	Sonic Hedgehog Signaling	1,75E+00
phagosome maturation	2,6	D-mannose Degradation	1,73E+00
Actin Nucleation by ARP-WASP Complex	2,57	Sertoli Cell-Sertoli Cell Junction Signaling	1,73E+00
Protein Ubiquitination Pathway	2,56	Role of CHK Proteins in Cell Cycle Checkpoint Control	1,72E+00
5-aminoimidazole Ribonucleotide Biosynthesis I	2,47	Glutaryl-CoA Degradation	1,70E+00
Thiosulfate Disproportionation III (Rhodanese)	2,47	BER pathway	1,70E+00
Glutathione Redox Reactions I	2,47	Mevalonate Pathway I	1,63E+00
VEGF Signaling	2,4	mTOR Signaling	1,62E+00
Huntington's Disease Signaling	2,29	ERK/MAPK Signaling	1,62E+00
Purine Nucleotides De Novo Biosynthesis II	2,28	CTLA4 Signaling in Cytotoxic T Lymphocytes	1,61E+00
Methylmalonyl Pathway	2,18	G Beta Gamma Signaling	1,61E+00
Proline Biosynthesis I	2,18	RhoA Signaling	1,57E+00
Paxillin Signaling	2,14	Cell Cycle Regulation by BTG Family Proteins	1,57E+00
LPS/IL-1 Mediated Inhibition of RXR Function	2,09	DNA Double-Strand Break Repair by Non-Homologous	1,57E+00
Germ Cell-Sertoli Cell Junction Signaling	2,08	Colanic Acid Building Blocks Biosynthesis	1,57E+00
Glycogen Degradation III	2,07	Regulation of Actin-based Motility by Rho	1,56E+00
Noradrenaline and Adrenaline Degradation	2,06	Atherosclerosis Signaling	1,54E+00
Rac Signaling	2,06	Germ Cell-Sertoli Cell Junction Signaling	1,52E+00
Neuregulin Signaling	2,01	Telomere Extension by Telomerase	1,51E+00
DNA Double-Strand Break Repair by Non-Homologous	1,97	Leukotriene Biosynthesis	1,51E+00
2-oxobutanoate Degradation I	1,97	Vitamin-C Transport	1,51E+00
Tight Junction Signaling	1,94	Xenobiotic Metabolism Signaling	1,50E+00
UDP-N-acetyl-D-glucosamine Biosynthesis II	1,81	Leukocyte Extravasation Signaling	1,49E+00
Glycogen Biosynthesis II (from UDP-D-Glucose)	1,81	Cellular Effects of Sildenafil (Viagra)	1,47E+00
Production of Nitric Oxide and Reactive Oxygen Species i	1,7	Methylglyoxal Degradation III	1,46E+00
p70S6K Signaling	1,7	Î²-alanine Degradation I	1,43E+00
Aryl Hydrocarbon Receptor Signaling	1,68	Proline Degradation	1,43E+00
Eicosanoid Signaling	1,68	L-DOPA Degradation	1,43E+00
Glucocorticoid Receptor Signaling	1,59	4-hydroxyproline Degradation I	1,43E+00
Glucose and Glucose-1-phosphate Degradation	1,55	GDP-L-fucose Biosynthesis I (from GDP-D-mannose)	1,43E+00
CTLA4 Signaling in Cytotoxic T Lymphocytes	1,53	Formaldehyde Oxidation II (Glutathione-dependent)	1,43E+00
Ethanol Degradation II	1,53	Glycine Biosynthesis I	1,43E+00
Mitochondrial Dysfunction	1,5	Superpathway of Geranylgeranyldiphosphate Biosynth	1,41E+00
Cellular Effects of Sildenafil (Viagra)	1,5	Paxillin Signaling	1,39E+00
Phospholipase C Signaling	1,5	Mechanisms of Viral Exit from Host Cells	1,39E+00
Acetyl-CoA Biosynthesis III (from Citrate)	1,47	Relaxin Signaling	1,39E+00
Melatonin Degradation III	1,47	Melatonin Signaling	1,38E+00
Sorbitol Degradation I	1,47	GPCR-Mediated Integration of Enteroendocrine Signalin	1,36E+00
D-mannose Degradation	1,47	RhoGDI Signaling	1,36E+00
Ephrin Receptor Signaling	1,46	Oxidative Ethanol Degradation III	1,32E+00
Prostanoid Biosynthesis	1,45		
UDP-N-acetyl-D-galactosamine Biosynthesis II	1,45		
Pyrimidine Deoxyribonucleotides De Novo Biosynthesis	1,43		
Ephrin B Signaling	1,43		
Axonal Guidance Signaling	1,4		
Tryptophan Degradation X (Mammalian, via Tryptamin	1,38		
Inhibition of Matrix Metalloproteases	1,38		
Xenobiotic Metabolism Signaling	1,35		
Neuroprotective Role of THOP1 in Alzheimer's Disease	1,34		

Supplementary Table 12. MicroLC-MSTrap targeted proteins/peptides and peptide specific information.

protein	peptide sequence	parent ion (m/z)	fragment 1 (m/z)	fragment 2 (m/z)	fragment 3 (m/z)
MDH2 A	ANTFVAELK	496,77	807,4611	706,4134	559,345
	H-ANTFVAEL(K_{C13N15})-OH	500,7813	815,4753	714,4276	567,3592
MDH2 B	VNPVIGGHAGK	383,2243	639,3573	526,2732	738,4257
	H-VNPVIGGHAG(K_{C13N15})-OH	385,8957	647,3715	534,2874	746,4399
KHSRP A	VQISPDSGGLPER	677,8517	927,453	715,3733	1014,485
	H-VQISPDSGGLPE(R_{C13N15})-OH	682,8558	937,4613	725,3816	1024,493
KHSRP B	IINDLLQSLR	592,8535	958,5316	729,4617	616,3777
	H-IINDLLQSL(R_{C13N15})-OH	597,8576	968,5399	739,47	626,386
DNM1L A	SSVLESLVGR	523,7957	773,4516	660,3675	531,3249
	H-SSVLESLVG(R_{C13N15})-OH	528,7998	783,4598	670,3758	541,3332
DNM1L B	SATLLQLITK	544,3397	715,4713	828,5553	929,603
	H-SATLLQLIT(K_{C13N15})-OH	548,3468	723,4855	836,5695	937,6172
OLA1 A	IGIVGLPNVGK	533,8343	783,4723	684,4039	514,2984
	H-IGIVGLPNVG(K_{C13N15})-OH	537,8417	791,4865	692,4181	522,3126
OLA1 B	IPAFLNVVDIAGLVK	784,9742	814,5033	1027,615	487,3239
	H-IPAFLNVVDIAGLV(K_{C13N15})-OH	788,9813	822,5175	1035,629	495,3381
