# Supplementary material for: Integrative proteomics in prostate cancer uncovers robustness against genomic and transcriptomic aberrations during disease progression
Source: Nat Commun. 2018 Mar 21;9:1176. doi: 10.1038/s41467-018-03573-6 (PMC5862881; doi:10.1038/s41467-018-03573-6)
Supplement: Supplementary file 3 — Description of Additional Supplementary Files(PDF 167 kb) [file 41467_2018_3573_MOESM3_ESM.pdf]

Description of Additional Supplementary Files

File Name: Supplementary Data 1
Description: Original mass spectrometry data.

File Name: Supplementary Data 2
Description: All proteins and number of unique peptides used in quantification of proteins.

File Name: Supplementary Data 3
Description: Genes regulated by differentially expressed miRNAs according to Mrna expression.

File Name: Supplementary Data 4
Description: Genes regulated by differentially expressed miRNAs according to protein expression.
